# Nucleolar expansion and elevated protein translation in premature aging

**DOI:** 10.1038/s41467-017-00322-z

**Published:** 2017-08-30

**Authors:** Abigail Buchwalter, Martin W. Hetzer

**Affiliations:** Molecular and Cell Biology Laboratory, The Salk Institute for Biological Studies, 10010 North Torrey Pines Road, La Jolla, CA 92037 USA

## Abstract

Premature aging disorders provide an opportunity to study the mechanisms that drive aging. In Hutchinson-Gilford progeria syndrome (HGPS), a mutant form of the nuclear scaffold protein lamin A distorts nuclei and sequesters nuclear proteins. We sought to investigate protein homeostasis in this disease. Here, we report a widespread increase in protein turnover in HGPS-derived cells compared to normal cells. We determine that global protein synthesis is elevated as a consequence of activated nucleoli and enhanced ribosome biogenesis in HGPS-derived fibroblasts. Depleting normal lamin A or inducing mutant lamin A expression are each sufficient to drive nucleolar expansion. We further show that nucleolar size correlates with donor age in primary fibroblasts derived from healthy individuals and that ribosomal RNA production increases with age, indicating that nucleolar size and activity can serve as aging biomarkers. While limiting ribosome biogenesis extends lifespan in several systems, we show that increased ribosome biogenesis and activity are a hallmark of premature aging.

## Abstract (web summary)

HGPS is a premature aging disease caused by mutations in the nuclear protein lamin A. Here, the authors show that cells from patients with HGPS have expanded nucleoli and increased protein synthesis, and report that nucleoli also expand as aging progresses in cells derived from healthy individuals.

## Introduction

Hutchinson-Gilford progeria syndrome (HGPS) is a fatal premature aging disorder caused by a sporadic, autosomal dominant mutation to a single gene encoding the nuclear protein lamin A^1^. Patients afflicted with this rare disease exhibit features of aging at a young chronological age, including loss of subcutaneous fat and hair, mobility deficits, and arteriosclerosis^2^. Complications of cardiovascular disease are fatal in the second decade of life.

A-type (A and C) and B-type (B1 and B2) lamins form a polymer meshwork that provides structural support to the nucleus^3^ and organizes the genome^4, 5^. In normal cells, lamin A is intricately processed after translation. Prelamin A is first farnesylated at its carboxy terminus^6^; a protease subsequently trims the carboxy terminus to generate mature lamin A. In HGPS, a synonymous mutation activates a cryptic RNA splice site in exon 11, resulting in substitution of ~ 50 amino acids near the carboxy terminus^1^. This substitution removes the protease cleavage site and generates a mutant form of lamin A, termed progerin, that is permanently lipid-modified^3^. Mutations to the lamin A protease, Zmpste24, cause a related but more lethal syndrome in humans termed restrictive dermopathy^7^, and Zmpste24 deletion causes an HGPS-like progeria in mice^8^. Since the same aberrant splicing event that generates progerin occurs with increasing frequency during normal aging^9–11^, progerin may contribute to both physiological and premature aging.

Progerin incorporates into the lamin scaffold and distorts nuclei^12^, disrupts the nucleoplasmic lamin meshwork^13^, depletes heterochromatin marks^14, 15^, sequesters nuclear proteins^16^, and induces DNA damage^17^. In addition to exhibiting defects to nuclear structure, cells derived from HGPS patients demonstrate systemic deficiences including elevated reactive oxygen species^16, 18^ and decreased cellular ATP^19, 20^. These cellular defects contribute to the exhaustion of mesenchymal stem cell pools, limiting tissue renewal^16, 21^. Importantly, many of these phenotypes are also observed in physiological aging, both at the cellular^9^ and tissue levels^22^.

While the defects associated with HGPS have been extensively described, the mechanistic link between progerin expression and aging remains unclear. The nuclear distortion induced by progerin expression^12^ suggests that HGPS may arise from accumulation of progerin protein. Similarly, inducing autophagy has been shown to decrease progerin levels and improve nuclear morphology, leading to the proposal that HGPS results from impaired progerin turnover^23, 24^. We therefore evaluated the effect of progerin on nuclear protein homeostasis. We report a profound shift in protein metabolism in HGPS, and provide evidence that progerin-induced disruption of nuclear organization activates nucleoli, allowing enhanced ribosome biogenesis, increased protein translation, and toxic depletion of intracellular energy stores. Further, we demonstrate that nucleolar size and activity increase with age in non-diseased individuals. These results identify nucleolar size as a novel aging biomarker and regulation of nucleolar activity as a possible therapeutic target.

## Results

### Accelerated protein turnover in HGPS patient fibroblasts

**Fig. 1:**
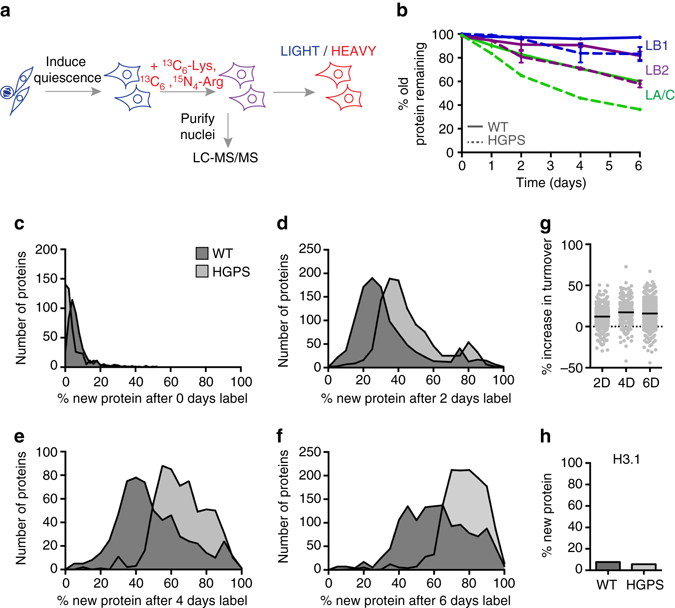
SILAC analysis of protein stability in HGPS. **a** Overview of SILAC experiment strategy in quiescent fibroblasts. Sub-confluent fibroblast cultures were cultured in low serum to induce quiescence for 3 days before initiating a 6-day ^13^C_6_-Lys, ^13^C_6_ / ^15^N_4_-Arg pulse-labeling timecourse. **b** Extent of lamin protein turnover quantified by % ^12^C_6_-Lys, ^12^C_6_ / ^14^N_4_-Arg-labeled proteins remaining (“old”) in WT cells (*solid lines*) and HGPS cells (*dashed lines*). LA/C, Lamin A/C; LB1, Lamin B1; LB2, Lamin B2. Mean ± SEM of all detected peptides shown. Peptide coverage was as follows: WT, LA/C 0D, *n* = 262; LA/C 1D, *n* = 41; LA/C 2D, *n* = 245; LA/C 4D, *n* = 140; LA/C 6D, *n* = 294. LB1 0D, *n* = 52; LB1 2D, *n* = 29; LB1 4D, *n* = 8; LB1 6D, *n* = 22. LB2 0D, *n* = 59; LB2 2D, *n* = 21; LB2 4D, *n* = 8; LB2 6D, *n* = 25. HGPS, LA/C 0D, *n* = 16; 1D, *n* = 114; 2D, *n* = 234; 4D, *n* = 80; 6D, *n* = 239. LB1 0D, *n* = 2; 1D, *n* = 7; 2D, *n* = 21; 4D, *n* = 6; 6D, *n* = 14. LB2 0D, *n* = 2; 1D, *n* = 9; 2D, *n* = 17; 4D, *n* = 1; 6D, *n* = 22. **c**–**f** Extent of protein turnover quantified by %protein labeled with ^13^C_6_-Lys and ^13^C_6_,^15^N_4_-Arg (“new”) after no label (**c**, 473 proteins); 2 days label (**d**, 1102 proteins), 4 days label (**e**, 603 proteins), or 6 days label (**f**, 1343 proteins). Turnover values were plotted for proteins detected by LC-MS/MS in both wild type (AG3258) and HGPS (AG11498) cells with at least two peptides at each time point shown. Distributions were significantly different at each time point (*p* < 0.0001); significance determined by unpaired *t*-test with Welch’s correction. **g** %increase in protein turnover in HGPS cells proteome-wide after 2 days labeling (median increase 12.1%), 4 days labeling (median increase 17.3%), or 6 days labeling (median increase 15.8%). *Black bars* indicate median. **h** %new Histone H3.1 detected after 2 days labeling in WT and HGPS cells based on the single tryptic peptide unique to H3.1. See also Supplementary Data 1

The lamina scaffolds the nucleus and plays a key role in establishing the nuclear proteome^25^. In proliferating cells, the nucleus and lamina are disassembled each time a cell divides. In non-dividing cells, turnover of the lamina is likely to occur by targeted removal and replacement of lamin subunits while the lamina remains largely intact. We sought to investigate the possibility that progerin alters protein homeostasis within the nucleus. To address this question, we performed a stable isotope labeling in cell culture (SILAC)^26^ time course in primary fibroblasts from HGPS patients and wild type (WT) parental controls (Fig. 1a, Supplementary Fig. 1a). In order to track protein turnover in the absence of cellular turnover, we induced cell cycle exit by culturing cells in low serum conditions (see Methods; Supplementary Fig. 1b). In WT human fibroblasts, we observed that A-type lamins turned over appreciably during the 6-day SILAC time course, while B-type lamins were very stable (Fig. 1b). We then analyzed lamin turnover rates in HGPS cells, where progerin is expressed along with normal A-type lamins (Supplementary Fig. 1a), although we did not detect the single tryptic peptide that distinguishes these isoforms by MS. We were surprised to find evidence for increased turnover of all lamin isoforms (lamins A/C, lamin B1, and lamin B2) in HGPS cells (Fig. 1b). This was surprising since our expectation was that progerin would be refractory to turnover. This finding then encouraged us to analyze turnover rates for the entire nuclear proteome and we found that, with few exceptions, most proteins turned over significantly more rapidly in HGPS-derived cells (Fig. 1c–f; see also Supplementary Data 1). At the midpoint of the time course (4 days), the proteome exhibited a median 17% increase in turnover compared to non-diseased cells (Fig. 1g). Importantly, Histone H3.1, which is not synthesized in quiescent cells^27^ incorporated little SILAC label in normal and HGPS samples (Fig. 1h), and neither cell population incorporated EdU when cultured in low serum conditions (Supplementary Fig. 1b) indicating that both cell populations maintain quiescence effectively. These data indicate a global shift in protein metabolism in HGPS.

### Global translation is increased in HGPS patient fibroblasts

**Fig. 2:**
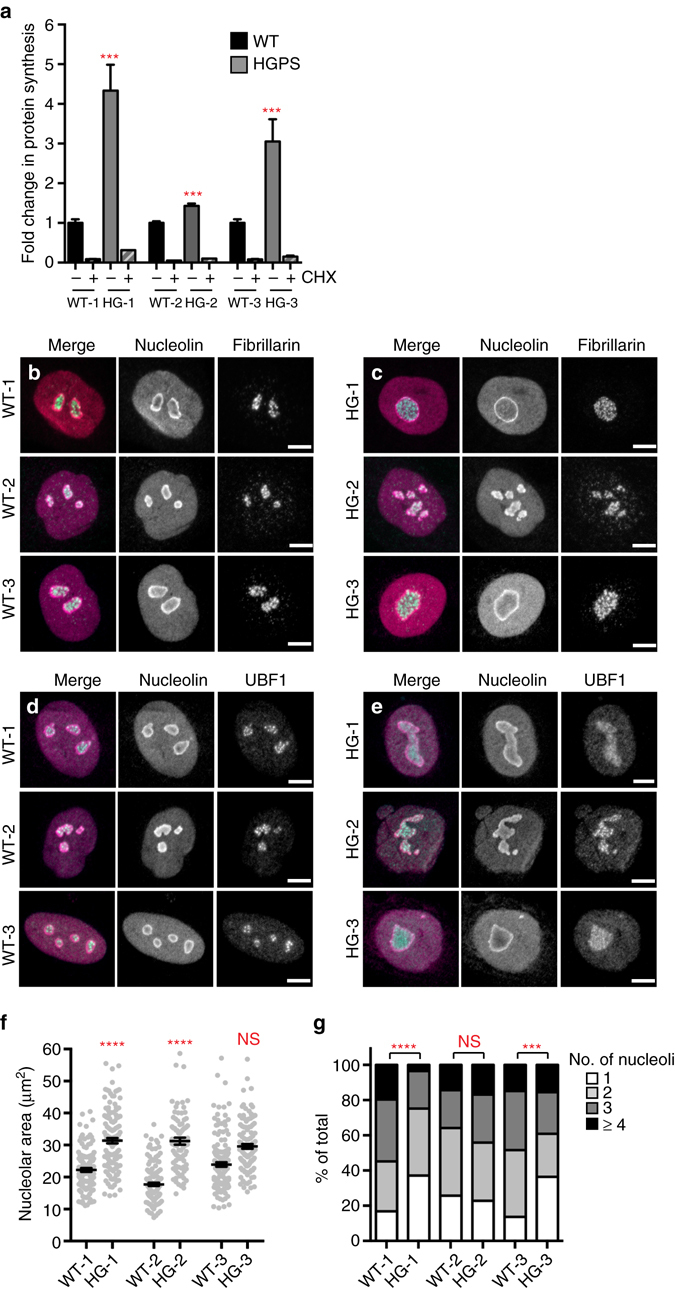
Global translation is increased and nucleoli are enlarged in HGPS. **a** Analysis of global translation rates by ^35^S-Met/Cys incorporation in WT and HGPS fibroblasts in the absence or presence of cycloheximide (CHX). Mean ± SEM of four independent experiments shown, each containing two technical replicates. *** indicates *p* < 0.001 by *t*-test. **b**–**e** Immunofluorescence of nucleolar proteins nucleolin (*magenta*) and fibrillarin (*cyan*) **b**, **c** or UBF1 (*cyan*) **d**, **e** in WT **b**, **d**) and HGPS **c**, **e** fibroblasts. *Scale bar*, 10 μm. **f** Total nucleolar cross-sectional area per cell, determined by boundaries of nucleolin immunofluorescence. **** indicates *p* < 0.0001 by *t*-test. *Bars* indicate mean ± SEM. **g** Number of nucleoli per cell. Significance determined by *χ* ^2^-test of indicated samples. **f**, **g** *N* > 111 cells per condition from six independent experiments with two technical replicates

The protein turnover measurements outlined above (Fig. 1) reflect the sum of two processes: clearance of old protein and de novo synthesis of new protein. All subsequent experiments were performed in cycling primary HGPS-derived cultures, to assess how protein metabolism is affected by progerin expression. We first assessed the status of protein degradation pathways in cycling HGPS cells and found no evidence for a global increase in protein catabolism by autophagy (Supplementary Fig. 2a, b) or proteasomal degradation (Supplementary Fig. 2c). In fact, proteasome activity was modestly decreased in HGPS cells, as has been previously reported^19^. We also confirmed that apoptosis was not induced in HGPS cells (Supplementary Fig. 2d). These data indicate that protein degradation pathways are not broadly activated in HGPS, and suggest that another process contributes to accelerated protein turnover. We therefore evaluated global protein synthesis rates in cycling cells by monitoring incorporation of radiolabeled amino acids, and found that translation is dramatically increased in all HGPS lines analyzed (Fig. 2a). From these findings we conclude that the increased turnover of the nuclear proteome observed in HGPS cells (Fig. 1) is due to elevated synthesis of new protein, and not to increased degradation of old protein. Consistently, polysome profiling indicated a higher proportion of ribosomes actively engaged in translation in cycling HGPS cells (Supplementary Fig. 3). Consistent with a previous report^28^, we find that HGPS cells cycle more rapidly than WT parental control cells (Supplementary Fig. 1c), which may be a means of coping with elevated protein load. This phase of increased protein production and cellular proliferation is not sustainable, however; HGPS cells senesce at earlier passages than WT cells do^28^.

Protein synthesis is controlled by signaling through the mammalian target of rapamycin (mTOR) pathway^29^. Intriguingly, suppressing mTOR signaling with rapamycin has shown promise in improving nuclear morphology of HGPS cells^23, 24^. Deletion of lamin A elevates mTOR signaling in mouse cardiac tissue^30^, suggesting that lamin A may influence mTOR signaling in some contexts. We, therefore, analyzed mTOR signaling in HGPS cultures. Phosphorylation of the direct mTOR targets 4EBP1 and eIF4G is unchanged in HGPS cells (Supplementary Fig. 4b, f), although it is possible that more total 4EBP1 is expressed (Supplementary Fig. 4a, e). Ribosomal protein S6 (RPS6) phosphorylation has complex effects on translation^27^ and can be catalyzed by S6 kinase, which is a direct mTOR target, or by other kinases. Levels of both RPS6 and phospho-RPS6 are consistently elevated in HGPS cells (Supplementary Fig. 4a, e), but mTOR-dependent S6 kinase phosphorylation is decreased (Supplementary Fig. 4b, e). Similarly, a previous analysis did not find evidence for mTOR pathway activation in HGPS cultures^23^, and emerging evidence indicates that lamin A’s influence on mTOR signaling in mouse models may be tissue-specific^31^. Overall, these data are not consistent with progerin increasing protein translation output by activating the mTOR pathway; rather, some alternative means of increasing protein synthesis must be involved. Since levels of the ribosome subunit RPS6 are elevated in HGPS cultures (Supplementary Fig. 4a), we investigated whether ribosome biogenesis is altered in HGPS cells.

### Enhanced ribosome biogenesis in HGPS patient fibroblasts

The nuclear structures responsible for ribosome biogenesis are nucleoli, which have characteristic tripartite organization and morphology that often correlates with activity^32, 33^. We analyzed markers of fibrillar centers (fibrillarin) and the dense fibrillar component (UBF1), which are bounded within the granular component, marked by nucleolin (Fig. 2b–e). Consistent with increased ribosome biogenesis in HGPS, we observed a general increase in total nucleolar area defined by nucleolin staining in HGPS cells, to a significant extent in 2 out of the 3 lines analyzed (Fig. 2f). This was accompanied by a decrease in nucleolar number in 2 out of the 3 lines analyzed (Fig. 2g). It thus appears that HGPS cells generally have fewer, but larger, nucleoli than WT cells. Fibrillarin and UBF1 organization within nucleoli appeared normal (Fig. 2b–e), indicating that these enlarged nucleoli are likely to be functional.

**Fig. 3:**
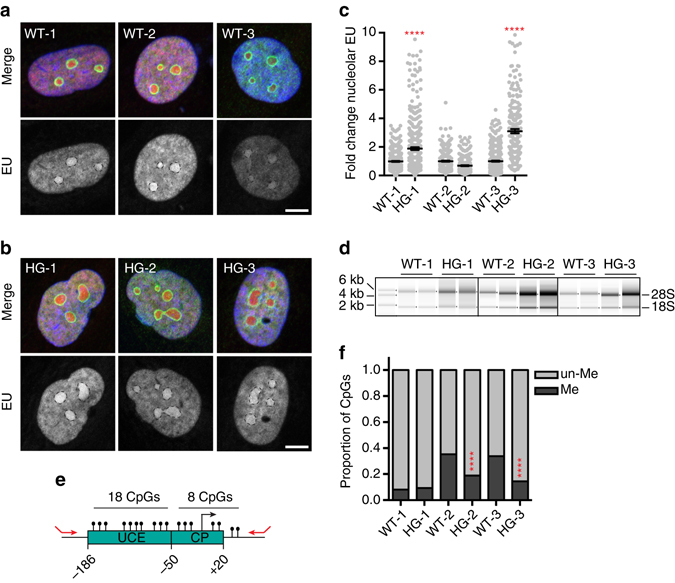
Increased rDNA transcription in HGPS. **a**, **b** Visualization of rRNA synthesis by a 4-h pulse of EU labeling (*magenta*) followed by immunostaining for nucleolin (*cyan*) in WT **a** or HGPS **b** fibroblasts. *Scale bar*, 10 μm. **c** Quantification of EU fluorescence intensity per nucleolus. **** indicates *p* < 0.0001 determined by *t*-test. *N* > 142 nucleoli analyzed per condition in two independent experiments with two technical replicates. *Black bars* indicate mean ± SEM. **d** Total RNA extracted from equal numbers of WT and HGPS fibroblasts. In all, 28 S (5 kb) and 18 S (1.9 kb) rRNAs are indicated. Representative images from one of three independent experiments shown. **e** Schematic map of the rDNA promoter. UCE, upstream control element; CP, core promoter. *Black knobs*, CpG methylation. In all, 18 CpGs in the UCE and 8 CpGs in the CP were analyzed. *Red arrows* indicate primers used for bisulfite sequencing. **f** Quantification of total CpG promoter methylation on rDNA promoters in WT and HGPS fibroblasts. See also Table 1. In 2 of 3 pairs, methylation is significantly decreased in HGPS (****, *p* < 0.0001, determined by *χ* ^2^-test). *N* > 270 CpGs analyzed per condition in two independent experiments

A nucleolus forms around a cluster of actively transcribing ribosomal DNA (rDNA) loci, of which there are ~ 300 in human cells distributed across five chromosomes^34^. At any given time, roughly half of the rDNA loci are silenced by dense CpG DNA methylation and repressive histone methylations, which antagonize promoter accessibility. Transcription from active rDNA loci constitutes the majority of cellular transcription^34^. Since widespread loss of heterochromatin is a hallmark of HGPS^14, 35^ (Supplementary Fig 1d, f), leading to inappropriate transcription through heterochromatic elements such as satellite DNA^14^, we hypothesized that nucleolar expansion in HGPS might be caused by de-repression of rDNA loci and thus increased production of ribosomal RNA (rRNA). To test this, we pulse-labeled nascent RNA using 5-ethynyl uridine (EU) and analyzed levels of nascent rRNA within nucleoli. We found increased transcription of rRNA in 2 of the 3 HGPS lines analyzed (Fig. 3a–c). This labeling disappears when Pol I is inhibited, as would be expected for Pol I-transcribed rRNA (Supplementary Fig. 5). Further, we evaluated abundance of the mature 28 S and 18 S ribosomal RNAs, and found that these rRNAs were more abundant in HGPS cells (Fig. 3d). Finally, we used bisulfite sequencing to determine the extent of CpG DNA methylation on the upstream control element (UCE) and core promoter (CP) regions of rDNA loci (Figs 3e, f; Table 1). In all lines analyzed, HGPS cells harbored a larger proportion of rDNA loci with completely un-methylated UCE and CP regions (Table 1, Supplementary Fig. 6). In 2 of the 3 HGPS lines analyzed, the density of CpG-methylated sites is significantly decreased along the UCE and CP regions on those rDNA loci that retain methylation (Fig 3f; Supplementary Fig. 6). Since Pol I binds selectively to rDNA repeats with un-methylated UCE and CP regions^36^, these data suggest that a larger proportion of rDNA repeats are engaged in transcription in HGPS. Overall, these data indicate that nucleoli are larger and more active in HGPS cells.

**Table 1:** Analysis of rDNA promoter methylation

Sample	No. of loci	No. of loci	% loci
	Me	un-Me	Me
WT-1	5	5	50.0
HG-1	3	7	30.0
WT-2	15	4	78.9
HG-2	14	6	70.0
WT-3	11	5	68.8
HG-3	7	8	46.7

Summary of number of rDNA loci analyzed and percentage of loci with any CpG methylation in WT and HGPS fibroblasts

**Fig. 4:**
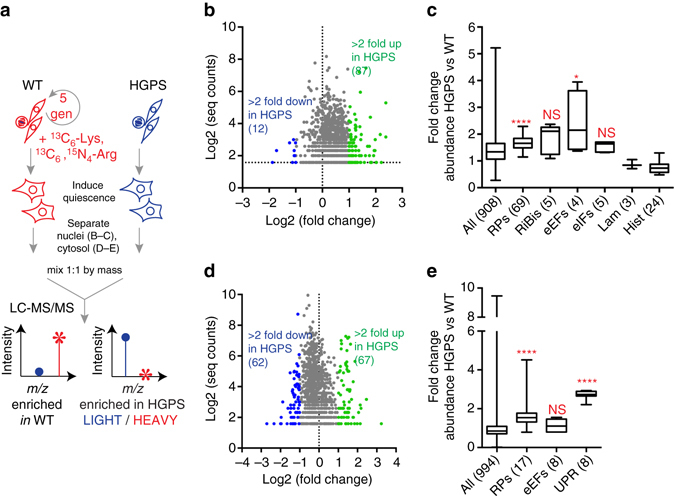
Elevated production of ribosomal proteins in HGPS. **a** Overview of SILAC abundance analysis strategy. Proliferating WT fibroblasts were cultured for five generations in media containing stable heavy isotopes to completely label cellular proteins. Parallel cultures of HGPS fibroblasts were expanded in normal media. Quiescence was induced; crude nuclear and cytosolic fractions were prepared, and equal masses of fractions were mixed and analyzed by LC-MS/MS. **b**, **d** Number of peptides detected vs. fold change in abundance on a logarithmic scale for **b** 908 proteins identified in nuclei and **d** 994 proteins identified in cytosol prepared from SILAC-labeled wild type (AG3258) and HGPS (AG11498) cells. **c**, **e**) Analysis of relative **c** nuclear and **e** cytosolic protein abundance by functional class. Subunits of the small and large ribosome (RPs); ribosome biogenesis proteins (RiBis); translation elongation factors (eEFs); translation initiation factors (eIFs); lamins (lam); histones (hist); UPR target genes (UPR). *Boxes* indicate 25th–75th percentile, *lines* indicate median values, and bars indicate range of values. Significance determined by Mann–Whitney *U*-test. See also Supplementary Data 2

Expansion of nucleoli and increased rRNA production are suggestive of increased ribosome biogenesis in HGPS. If this were the case, the production of other ribosomal proteins (RPs) in addition to RPS6 (Supplementary Fig. 4a, b) would also be increased. Since ribosome biogenesis takes place within the nucleus, we used SILAC to determine relative protein abundance^26^ in nuclear extracts prepared from non-dividing wild type vs. HGPS fibroblasts (Fig. 4a). Of the 908 proteins identified, 87 proteins were upregulated more than twofold, while only 12 proteins were downregulated more than twofold, and ~ 800 proteins differed less than twofold between the two populations (Fig. 4b, Supplementary Data 2). We detected 69 of the 80 known components of the 40 S and 60 S ribosome subunits in our analysis; as a group, RPs were significantly upregulated in the nuclei of HGPS cells (Fig. 4c, *p* < 0.0001, determined by Mann–Whitney test; see also Supplementary Data 2), consistent with increased ribosome biogenesis. *Trans-*acting factors involved in ribosome biogenesis^37, 38^, in particular nucleophosmin, fibrillarin, and NOP58, were also modestly increased in HGPS cells (Fig. 4c, Supplementary Data 2). In addition, translation elongation factors (eEFs) were more abundant in HGPS cells (Fig. 4c, *p* < 0.05, determined by Mann–Whitney test), which may also promote more efficient translation. Consistent results were observed when nuclear extracts from a second pair of WT and HGPS cells were analyzed in this way (Supplementary Fig. 7, Supplementary Data 3).

RPs are abundant in both the nuclear and cytosolic compartments, where they participate in ribosome biogenesis and protein translation, respectively. To analyze abundance of fully assembled ribosomes, we also determined relative protein abundance in cytosolic extracts prepared from wild type vs. HGPS fibroblasts. Of the 994 proteins identified, we detected 17 RPs that were significantly upregulated in the cytosol of HGPS cells (Fig. 4e, *p* < 0.0001, determined by Mann–Whitney test; see also Supplementary Data 2). eEFs were not significantly upregulated in the cytosol of HGPS cells (Fig. 4e). Altogether, these data indicate that increased production of both the nucleic acid and protein components of ribosomes sets the stage for increased protein translation in HGPS.

### Progerin expression causes nucleolar expansion

**Fig. 5:**
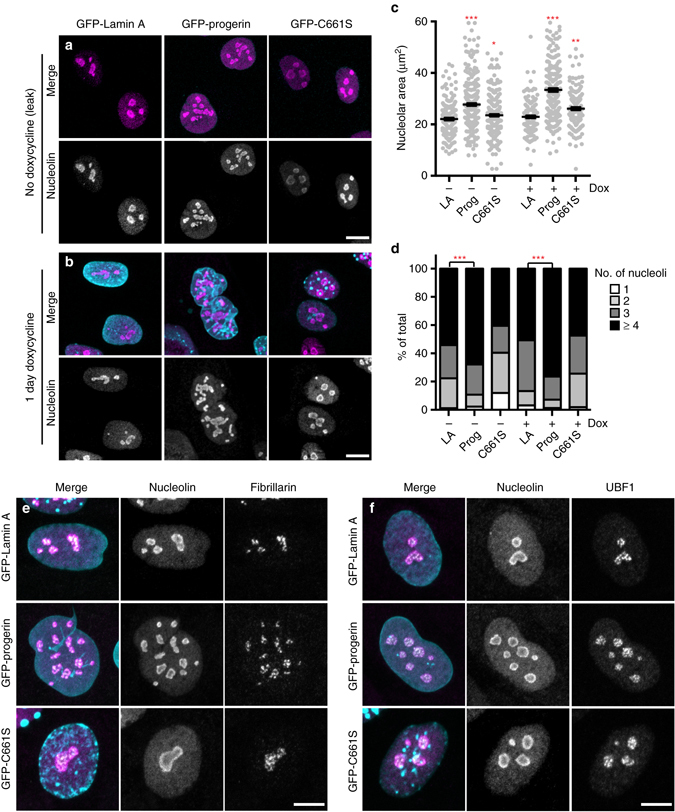
Progerin expression drives nucleolar expansion. **a**, **b** Human fibroblasts expressing GFP-tagged lamin A, progerin, or progerin C661S (*cyan*) under a doxycycline-inducible promoter stained for nucleolin (*magenta*) either in absence of doxycycline **a** or after 1-day treatment with doxycycline **b**. *Scale bar* = 10 μm. **c** Total nucleolar area per cell, determined by boundaries of nucleolin immunofluorescence. Significance determined by *t*-test. *Black bars* indicate mean ± SEM. **d** Number of nucleoli per cell. **c**, **d** *N* > 70 cells per condition from three independent experiments with two technical replicates. Significance determined by *χ* ^2^-test. **e**, **f** Staining for nucleolin (*magenta*) and fibrillarin (*gray*) **e** or UBF1 (*gray*) **f** in cells expressing the indicated GFP-tagged lamin proteins (*cyan*) after 1 day of doxycycline induction. *Scale bar* = 10 μm

To establish a causal link between expression of progerin and altered nucleolar architecture, we made use of immortalized human fibroblast cell lines that express GFP-tagged lamin A variants under a doxycycline-responsive promoter^15^ Supplementary Fig. 8. We assessed nucleolar size in cells expressing GFP-tagged lamin A, progerin, or progerin-C661S, which cannot be lipid-modified and does not exhibit progeroid phenotypes^15^. Strikingly, cells expressing GFP-tagged progerin, but not wild type lamin A or progerin-C661S, exhibited enlarged (Fig. 5a–c) and more numerous (Fig. 5d) nucleoli, suggesting that progerin expression is sufficient to promote nucleolar expansion. The tripartite organization of nucleoli was preserved in progerin-expressing cells in spite of the rapid expansion of the nucleolus (Fig. 5e, f). Nucleolar expansion was detectable even when GFP-progerin was expressed at low levels in the absence of doxycycline (Fig. 5a, c), and became more pronounced within 24 h of doxycycline treatment (Fig. 5b, c). Administering anti-GFP RNAi to further diminish leaky expression of GFP-progerin decreased nucleolar numbers to wild-type levels (Supplementary Fig. 9). Notably, nucleolar expansion occurred more rapidly and at lower expression levels than other progerin-dependent phenotypes reported in this system, including loss of the nucleoplasmic lamin A-binding protein LAP2α^15^ (Supplementary Fig. 10).

### Lamin depletion causes nucleolar expansion

**Fig. 6:**
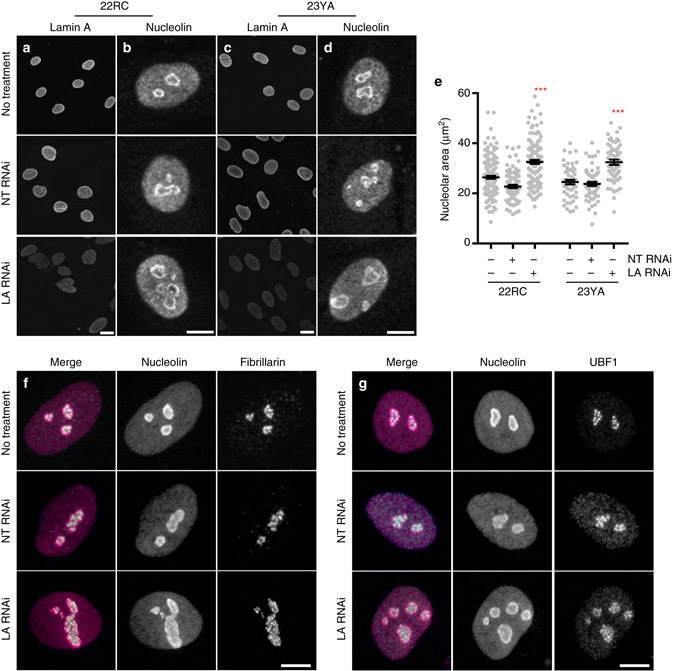
Lamin A depletion drives nucleolar expansion. **a**–**e** Normal human fibroblasts (22RC and 23YA) transfected as indicated with non-targeting (NT) or Lamin A/C-targeting (LA) RNAi for 72 h before staining for Lamin A/C **a**, **c** or nucleolin **b**, **d**. *Scale bar* = 20 μm **a**, **c**; 10 μm **b**, **d**. **e** Total nucleolar area per cell, determined by boundaries of nucleolin immunofluorescence. Significance determined by *t*-test. *Black bars* indicate mean ± SEM for *N* > 52 cells per condition from two independent experiments with two technical replicates. **f**, **g** Normal human fibroblasts (22RC) transfected as indicated with NT or LA RNAi for 72 h before staining for nucleolin (*magenta*) and fibrillarin (*cyan*) **f** or UBF1 (*cyan*) **g**. *Scale bar* = 10 μm

Wild type lamin A is a prominent component of the nuclear periphery but is also found within the nucleoplasm, at intranuclear foci^39^ and adjacent to nucleoli^40^. This nucleoplasmic pool of lamin A interacts with broad regions of both euchromatin and heterochromatin^41^. Progerin expression disrupts the nucleoplasmic pool of lamin A^13^ and causes nucleolar expansion (Fig. 5). Altogether this raises the exciting possibility that nucleoplasmic lamin A has a role in regulating nucleolar organization in normal cells. To test this, we treated primary human fibroblasts with RNAi targeting lamin A/C (Fig. 6a, c) and found that its depletion resulted in the expansion of nucleoli (Fig. 6b, d, e) but did not alter nucleolar numbers (data not shown). The organization of fibrillarin and UBF1 within nucleoli was not changed by LA knockdown, suggesting that nucleoli remain functional in the absence of LA (Fig. 6f, g). These findings delineate an unexpected connection between the nuclear lamina, nucleolar organization, and regulation of protein synthesis output.

### Nucleolar size and activity increase during normal aging

**Fig. 7:**
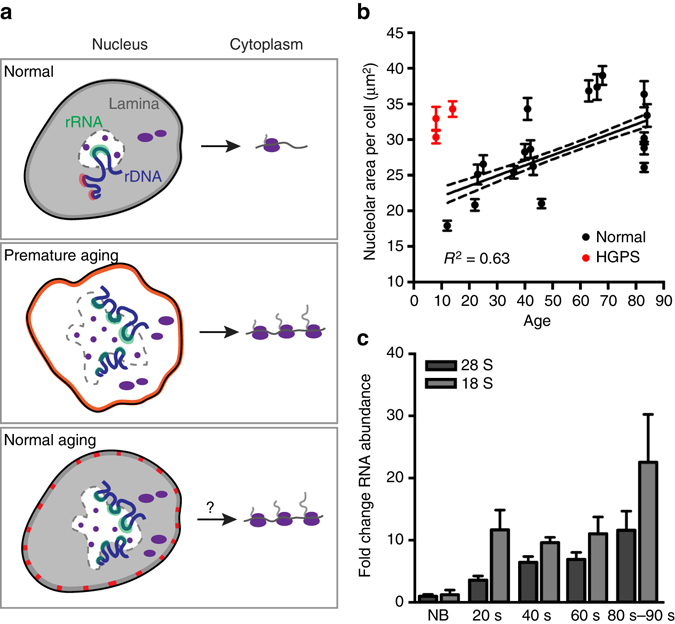
Nucleolar size and rRNA production increase during normal aging. **a** Total nucleolar area per cell, determined by boundaries of nucleolin immunofluorescence, in cells from healthy individuals of varying ages (*black*) or cells from HGPS patients (*red*). *Dots* and *bars* indicate mean ± SEM for *N* > 34 cells per condition from 1–2 independent experiments with two technical replicates. Pearson correlation coefficient 0.6, *p* < 0.005. See also Supplementary Fig. 11 and Supplementary Table 4. **b** Abundance of 28 S and 18 S rRNAs determined by qPCR in 2–3 samples each from healthy individuals of varying ages. Normalized to GAPDH mRNA. NB, newborn. *Bars*, SEM. Representative data from 1 of 2 independent experiments shown. See also Supplementary Fig. 12 and Supplementary Table 5. **c** Model. In normal cells (*top panel*), the nucleoplasmic lamin network represses nucleolar activity. In HGPS (*middle panel*), the nucleoplasmic lamin network is lost. Loss of rDNA repression leads to elevated rRNA transcription, enlarged nucleoli, increased expression of ribosomal proteins, and increased translation output. In normal aging (*bottom panel*), progerin is sporadically expressed and incorporates into the lamina (*red*). Nucleoli expand, produce more rRNA, and possibly produce more translating ribosomes

Diminishing protein translation either by removing ribosomal subunits^42, 43^ or by pharmacologically interfering with translation^44^ has the beneficial effect of extending lifespan. At the other extreme, our findings implicate expanded nucleoli and runaway protein translation in premature aging. To test whether nucleolar size correlates with progression of physiological aging, we analyzed nucleolar size in a panel of primary human fibroblasts from healthy donors ranging from 12 to 84 years of age (Fig. 7b, Supplementary Fig. 11). We found a significant and direct correlation between aging and nucleolar size in healthy individuals. Conversely, the Antebi laboratory has shown that small nucleoli predict longevity in *C. elegans* ^45^. Cells derived from HGPS patients are extreme outliers from the trend observed for healthy individuals (Fig. 7a), exhibiting nucleoli of comparable size to elderly individuals, in spite of their origin from donors between 8 and 14 years of age. Further, we quantified levels of 28 S and 18 S rRNAs in healthy donor fibroblasts and found that old cells have a higher rRNA content (Fig. 7c, Supplementary Fig. 12), suggesting that enlarged nucleoli are more active in generating rRNAs in aged cells. We speculate that de-repression of rDNA transcription occurs as a consequence of diminishing robustness of heterochromatic marks^9, 46^ and/or as a consequence of sporadic progerin expression^10, 11, 47^ during normal aging (Fig. 7a). Altogether, our data indicate that increased nucleolar size and activity are hallmarks of aging in both pathological and physiological contexts.

## Discussion

Our data are consistent with the model (Fig. 7a) that progerin acts as a dominant negative mutant to prevent the previously unappreciated role of lamin A in organizing nucleoli and limiting ribosome biogenesis. Since both lamin A depletion (Fig. 6) and ectopic expression of progerin (Fig. 5) rapidly induce nucleolar expansion, it is likely that the lamin network is directly involved in regulating nucleolar activity.

Progerin depletes the nucleoplasmic lamin A meshwork^13^, which normally interacts with large domains of both euchromatin and heterochromatin^5^. We propose that as progerin disrupts the global heterochromatin environment^12, 14^ (Supplementary Fig. 1d, f), repressive marks normally found on inactive rDNA loci are also lost. We have shown that a larger proportion of rDNA promoters are un-methylated in HGPS (Fig. 3f, Table 1), and thus are in the chromatin state that allows Pol I binding^36^. Changes to the methylation status of rDNA in HGPS have not been previously reported. However, rDNA methylation was analyzed in the *Zmpste24 −/−* mouse, which models many features of HGPS^48^. rDNA loci in *Zmpste24 -/-* animals exhibited higher methylation, particularly within the rDNA gene body. It is possible that analysis of rDNA methylation in *Lmna* ^*G608G/G608G*^ knock-in mice, which harbor the HGPS allele^49^, may be more consistent with the results we observe in human HGPS cultures. Separately, recent work indicates that murine and human rDNA repeats may be regulated by distinct mechanisms^34^. In addition to regulating transcriptional output, heterochromatin maintains the stability of repetitive elements, including rDNA^50, 51^. It is possible that loss of heterochromatin marks on rDNA loci in HGPS may lead to recombination, insertions and/or deletions of rDNA repeats. Indeed, HGPS cells exhibit features of genomic instability and pervasive DNA damage^17^.

We conclude that rDNA de-repression leads to increased rRNA transcription in HGPS, based on the visualization of increased nucleolar transcription (Fig. 3a–c), and the increased abundance of mature 28 S and 18 S rRNAs (Fig. 3d). One would also expect to see increased abundance of immature pre-rRNA species, although we did not assess this. We also observe nucleolar expansion, consistent with increased nucleolar rRNA production (Fig. 2b–f). Since transcription of rDNA generates a large proportion of total cellular RNA, overproduction of rRNA may be an emergent property of the general heterochromatin loss that occurs in HGPS or with the progression of physiological aging.

Since rRNA and RP production are coordinately regulated^52^, rRNA production can in turn induce production of RPs. We also observe elevated production of RPs in HGPS (Fig. 4, Supplementary Fig. 7), which assemble into functional polysomes (Supplementary Fig. 3), in the end increasing global translation rates (Fig. 2a). Ribosome biogenesis and protein synthesis are enormously energy-expensive, together demanding roughly 80% of the cell’s ATP by some estimates^53^. We surmise that over-production of protein in HGPS may promote premature aging by depleting cellular energy stores. Consistently, HGPS-derived cells are ATP-depleted^19, 20^, and patients afflicted with HGPS exhibit an elevated metabolic rate^54–56^. We find evidence (Fig. 4e) for upregulation of the unfolded protein response (UPR) in HGPS fibroblasts, suggesting that HGPS cells must upregulate folding chaperones to cope with increased protein load.

We do not yet know how quiescent and proliferating cell types are affected by progerin-driven de-regulation of protein synthesis in vivo. Consistent with a previous report^28^, we observe that HGPS cells cycle more rapidly at early passages, possibly in response to elevated protein synthesis rates (Supplementary Fig. 1c). We first observed indirect evidence of increased protein synthesis in non-dividing cells (Fig. 1), but how non-dividing cells cope with elevated protein synthesis remains unclear. It is clear, however, that changes to the function of both quiescent and proliferating cell populations each contribute to age-associated decline of tissues and organs^57^. Non-dividing cell types are especially vulnerable to accumulation of protein and DNA damage over time. Separately, the ability of stem cell pools to proliferate in order to repair tissue damage declines with age^58^, and replicative senescence of cells in proliferative tissues also contributes to aging^59^. Importantly, models of HGPS exhibit both stem cell exhaustion^21^ and accelerated senescence onset^60^. We anticipate that de-regulation of ribosome biogenesis and protein synthesis by progerin expression profoundly affects cellular energy metabolism in both non-dividing and dividing cell states and contributes to organismal aging.

It seems likely that interventions that limit ribosome biogenesis or translation increase lifespan by counteracting age-linked upward drift in these processes. Consistent with this idea, we report that nucleolar size increases with age in humans (Fig. 7b). Previous work in *S. cerevisiae* indicates that nucleolar fragmentation^61^ and accumulation of rDNA circles^62^ increase with age, and increased production of much of the proteome, especially RPs, has been observed in aged *C. elegans* ^63^. We do not currently know whether protein synthesis is similarly increased during human aging. Conversely, protein turnover is slower in long-lived *daf-2* mutant worms^64^, and nucleolar size and activity are decreased in a variety of long-lived mutant backgrounds^45^.

Since mTOR regulates both ribosome biogenesis^52^ and translation initiation^29^, inhibition of the mTOR pathway could alleviate phenotypes of HGPS^23^ by pushing back against the influence of progerin on ribosome biogenesis and translation. While modulation of the mTOR pathway by rapamycin and analogs has shown promise in extending lifespan^44^ and reversing cellular phenotypes of HGPS^23, 24^, enthusiasm has been tempered by the possibility of side effects arising from prolonged treatment^65^. Our findings imply that direct inhibition of rRNA production, perhaps using drugs that are currently in the trial pipeline for other diseases^66^ could be an attractive target for treatment of HGPS and possibly for extension of human lifespan.

## Methods

### Reagents and antibodies

Goat anti-lamin A/C N-18(sc-6215), rabbit anti-LAP2a(sc-28541), and mouse anti-UBF1(sc-13125) were purchased from Santa Cruz Biotechnology. Nucleolin(#14574), phospho (Ser 235/236) S6(#4858), Histone H3(#4499), LC3B(#3868), PARP(#9532), 4EBP1(#9644), S6, phospho(Ser 1108) eIF4G(#2441),, and phospho (Ser65) 4EBP1(#9451) rabbit antibodies were purchased from Cell Signaling. Mouse anti-fibrillarin(NB300-269) was purchased from Novus. Rabbit anti-H3K9me3(ab8898) was purchased from Abcam. ^13^C_6_-Lysine and ^13^C_6_,^15^N_4_-Arginine were purchased from Sigma-Aldrich.

### Cell culture

Primary human dermal fibroblasts AG16409, AG04386, AG04457, AG05413, AG02974, AG05274, AG3257, AG3258, AG22153, AG21753, AG05247, AG10884, and AG07725 (wild type) and AG11513, AG11498 (HGPS, *LMNA* G608G splice site mutation) were obtained from Coriell Cell Research (https://catalog.coriell.org). Human dermal fibroblasts HGFDFN168 (wild type) and HGADFN167 (HGPS, *LMNA* G608G splice site mutation) were obtained from the Progeria Research Foundation. In this work, pairs of WT and HGPS fibroblasts are referred to as follows: WT-1, AG3258; HGPS-1, AG11498; WT-2, AG3257; HGPS-2, AG11513; WT-3, HGFDFN168; HGPS-3, HGADFN167. WT-3 is an unaffected parent of HGPS-3; pairs 1 and 2 are unrelated. Expression of mutant lamin A in HGPS lines was verified (Supplementary Fig. 1a). See Supplementary Tables 1 and 2 for specific cell lines used in experiments that appear in Fig. 7a, b, respectively. Human dermal fibroblasts 22RC, 23 VA, 25MH, 62MP, 64JW, and 67LR were prepared from 4 mm skin biopsies obtained from human donors. Informed consent was obtained in writing from all donors in accordance with the Salk Institute Internal Review Board Protocol for obtaining human samples. See Supplementary Tables 1 and 2 for donor age and sex information. Biopsies were dissected and placed in growth medium containing collagenase for 1 h at 37 °C. Samples were resuspended in growth medium and distributed to gelatin-coated plates. Fibroblast cultures began to grow out from tissue approximately 1 week after seeding.

All cellular assays were performed at passage <20. Cells were grown in MEM supplemented with 15% FBS, non-essential amino acids, and antibiotics.

Immortalized human fibroblasts expressing GFP-lamin A, GFP-progerin, and GFP-progerin C661S under a doxycycline-responsive promoter were provided by the Misteli laboratory^15^. Doxycycline levels were optimized for each cell line to give roughly equivalent levels of GFP fusion protein expression as described;^15^ GFP-LA was induced with 100 ng/ml doxycycline, GFP-progerin-C661S with 8 ng/ml doxycycline, and GFP-progerin with 500 ng/ml doxycycline.

### RNA interference

An siRNA oligonucleotide sequence targeting human Lamin A/C was used with the following sequence: UGU UCU UCU GGA AGU CCA GTT. A Sigma Mission Universal Negative Control siRNA (SIC001, Sigma-Aldrich) was used as a negative control. Cells were transfected with 50 nM RNAi using siLentFect transfection reagent (Bio-Rad) according to the manufacturer’s instructions. Cells were incubated for 72 h after RNAi transfection before processing for analysis.

### Preparation of SILAC-labeled proteins

Fibroblasts were induced to enter quiescence by switching subconfluent cultures to growth media containing 1% serum for 3–5 days. For pulse-labeling analyses, quiescent fibroblasts were switched to media containing stable heavy isotopes of lysine and arginine (^13^C_6_-Lysine, ^13^C_6_, ^15^N_4_-Arginine) for 1 to 6 days. Media was refreshed every other day. For protein abundance analyses, cycling wild type fibroblasts were grown for five population doublings in media containing stable heavy isotopes of lysine and arginine^26^. A parallel population of HGPS fibroblasts were grown in normal media. Both populations were induced to enter quiescence for 5 days before harvesting for SILAC.

Crude nuclear extracts were prepared similarly to the method of Schirmer et al.^67^., as follows. Cells were harvested in PBS, then swollen in hypotonic lysis buffer (10 mM potassium acetate, 20 mM Tris acetate pH 7.5, 0.5 mM DTT, 1.5 mM MgCl_2_, and protease inhibitors), followed by mechanical lysis through a 25-gauge needle and syringe. The nuclei were pelleted and the supernatant retained for cytosolic fractions. Nuclei were then resuspended in buffer containing 10 mM Tris pH 8.0, 10% sucrose, 1 mM dithiothreitol (DTT), 0.1 mM MgCl2, 20 ug/ml DNase I, and 1 ug/ml RNase I. After nuclease treatment, nuclei were layered on top of a 30% sucrose cushion and pelleted. For pulse-labeled SILAC samples, crude nuclei were extracted in 10 mM Tris pH 8, 1% *n*-octyl glucoside, 400 mM NaCl, and 1 mM DTT, and extracts and pellets were prepared separately for liquid chromatography–mass spectrometry (LC-MS)/MS. For abundance analysis SILAC, protein content of the fractions was determined by BCA assay. Equal protein masses of wild type and HGPS extracts were mixed 1:1 before trypsin digestion and protein identification by LC-MS/MS.

### Mass spectrometry

Samples were denatured in 8 M urea/100 mM TEAB, pH 8.5; reduced with TCEP; alkylated with chloroacetamide; and digested overnight with trypsin. Digestion was quenched with 5% formic acid. Detergent was removed from pulse-labeled SILAC samples with SCX tips. Samples corresponding to SILAC analyses appearing in Fig. 1, Fig. 4d, e, and Supplemental Fig. 7 were run on a Thermo Orbitrap Fusion Tribrid MS/MS with CID fragmentation. The digest was injected directly onto a 30 cm, 75 um ID column packed with BEH 1.7 um C18 resin. Samples were separated at a flow rate of 200 nl/min on a nLC 1000. Buffer A and B were 0.1% formic acid in water and acetonitrile, respectively. A gradient of 1–25%B over 160 min, an increase to 35%B over 60 min, an increase to 90%B over another 10 min and held at 90%B for a final 10 min of washing was used for 240 min total run time. Column was re-equilibrated with 20 ul of buffer A prior to the injection of sample. Peptides were eluted directly from the tip of the column and nanosprayed directly into the mass spectrometer by application of 2.5 kV voltage at the back of the column. The Orbitrap Fusion was operated in a data dependent mode. Full MS^1^ scans were collected in the Orbitrap at 120 K resolution with a mass range of 400 to 1500 *m*/*z* and an AGC target of 4e^5^. The cycle time was set to 3 s, and within this 3 s the most abundant ions per scan were selected for CID MS/MS in the ion trap with an AGC target of 1e^4^ and minimum intensity of 5000. Maximum fill times were set to 50 ms and 100 ms for MS and MS/MS scans respectively. Quadrupole isolation at 1.6 *m*/*z* was used, monoisotopic precursor selection was enabled, charge states of 2–7 were selected and dynamic exclusion was used with exclusion duration of 5 s.

Samples corresponding to the SILAC analysis appearing in Fig. 4b, c were run on a Thermo Q-Exactive Quadrupole-Orbitrap MS/MS using previously optimized settings^68^. The digest was injected directly into a 30 cm, 75 um ID column packed with BEH 1.7 um C18 resin. Samples were separated at a flow rate of 200 nl/min on an LC 1000. Buffer A and B were 0.1% formic acid in water and acetonitrile, respectively. A gradient of 5–30% B over 280 min, an increase to 40% B over 60 min, and increase to 90% B for another 10 min, followed by 90% B for the final 10 min was used for the 360 min total run time. Peptides were eluted directly from the tip of the column and nanosprayed directly into the mass spectrometer by application of 2.5 kV voltage at the back of the column. The Q Exactive was operated in data-dependent mode. Full MS1 scans were collected in the Orbitrap at 70 k resolution with a mass range of 400 to 1800 *m*/*z* and an AGC target of 5e^6^. The ten most abundant ions per scan were selected for MS/MS analysis with HCD fragmentation of 25NCE, an AGC target of 5e^6^ and minimum intensity of 1e^4^. Maximum fill times were set to 120 ms and 500 ms for MS and MS/MS scans respectively. Quadrupole isolation of 2.0 *m*/*z* was used, dynamic exclusion was set to 15 s and charge states of 1 and unassigned were excluded.

Peptide and protein identification, quantification, and analysis were performed with Integrated Proteomics Pipeline-IP2 (Integrated Proteomics Applications; www.integratedproteomics.com). Tandem mass spectra were extracted from raw files using RawConverter^69^ and searched with ProLUCID^70^ against the human UniPROT database. The search space included all fully tryptic and half-tryptic peptide candidates. Carbamidomethylation on cysteine was considered as a static modification. Data were searched with 50 ppm precursor ion tolerance and 600 ppm fragment ion tolerance. Data was filtered to 10 ppm precursor ion tolerance post-search. Identified proteins were filtered using DTASelect^71^ and utilizing a target-decoy database search strategy to control the false discovery rate to 1% at the protein level. Census^72^ was utilized for quantitative analysis of SILAC labeled peptides. Peptide ratios were calculated for each tryptic peptide with a profile score >0.8 as the peak area ratio of the heavy isotope-containing mass spectrum to light isotope-containing mass spectrum. Ratios were averaged for all peptides identified for each protein.

### Preparation of cell lysates and western blotting

Equal numbers of cells were lysed in PBS/1% Tx100 / 0.1% SDS supplemented with complete protease inhibitor cocktail (Roche) and phosSTOP phosphatase inhibitor (Roche). Cells were lysed by 10 passages through a 25-gauge needle. Equal volumes of whole-cell lysate were run on acrylamide gels, transferred to nitrocellulose, blotted with primary and secondary antibodies diluted in 5% BSA in TBST, and quantified using a fluorescent imager (Odyssey; Li-Cor). Primary antibodies were diluted 1:1000; secondary antibodies were diluted 1:5000. The integrated density of the bands of interest were normalized to Histone H3 signal prior to comparison across conditions.

### Immunofluorescence

Cells were fixed in 4% PFA for 5 min and permeabilized and blocked in IF buffer (PBS, 0.1% Tx100, 0.02% SDS, 10 mg/ml BSA) before staining with primary and secondary antibodies diluted in IF buffer. Primary antibody dilutions were as follows. Nucleolin, 1:500; fibrillarin, 1:250; UBF1, 1:250; Lamin A, 1:500; H3K9me3, 1:500. All secondary antibodies were diluted 1:1000. Coverslips were mounted in ProLong Gold (Life Technologies) and imaged on a Zeiss LSM 710 scanning confocal microscope with a 63 × 1.4 NA objective. Images are shown as maximum intensity projections of z series. Nucleolar size was quantified by measuring the cross-sectional area occupied by a mask corresponding to nucleolin stain; all image quantification was performed with ImageJ.

### RNA labeling

EU pulse labeling of RNA was performed according to the manufacturers’ instructions (Click-IT EU RNA labeling kit, Life Technologies). Cells on coverslips were incubated in growth medium containing 1 mM EU for 4 h, fixed, stained for EU, and immunostained for nucleolin. For Pol I inhibition, EU incubation was performed in medium supplemented with 0.1 ug/ml actinomycin D to specifically inhibit Pol I transcription^73^. EU intensity was quantified from images acquired at the same laser power and gain settings by measuring the total signal intensity in the region occupied by a mask corresponding to the nucleolin stain.

### RNA electrophoresis

For analysis of total cellular RNA, RNA was prepared from 2.5 x 10^5^ cells using the RNeasy RNA Mini Kit (Qiagen) according to the manufacturer’s instructions. Equal volumes of pure RNA were analyzed by capillary electrophoresis on a TapeStation system (Ambion). See Supplementary Fig. 13 for uncropped images corresponding to the data shown in Fig. 3.

### Radiolabeling

Cells seeded in 35 mm dishes were washed once in Met/Cys depleted media, then incubated with Met/Cys depleted media supplemented with 150 μCi ^35^S-Met/Cys EasyTag Express (Pierce) for 45 min at 37 °C. As a control, some cells were pre-treated with cycloheximide for 2 h to block protein translation, then incubated with ^35^S label in the presence of cycloheximide. Cells were washed in PBS, lysed in PBS/1% Tx100/0.1% SDS, and protein content determined by BCA assay. Twenty microgram of total protein was spotted onto filters and precipitated with 5% and 10% TCA washes. Filters were then washed in ethanol and acetone, and dried in scintillation vials. ^35^S content was determined by scintillation counting.

### Polysome profiling

Cells were incubated in growth medium with 100 μg/ml cycloheximide for 3 min before washing 3× in cold PBS supplemented with 100 μg/ml cycloheximide, then collecting by scraping in PBS with cycloheximide. Cells were lysed in polysome extraction buffer (10 mM Tris pH 7.4, 150 mM NaCl, 15 mM MgCl2, 0.5% Triton-X-100, 100 μg/ml cycloheximide, 0.5 mg/ml heparin) (PEB), then clarified by centrifugation at 12,000×*g* for 10 min. Supernatants were normalized by absorbance at 260 nm before loading onto the top of a 10–50% sucrose gradient. Sucrose gradients were prepared fresh by dissolving sucrose in PEB without Triton-X-100. Gradients were spun at 35,000 rpm at 4 C for 150 min in an SW41 rotor. Gradients were analyzed by collecting fractions through a ultraviolet detector at 254 nm.

### Bisulfite mapping

Genomic DNA was extracted from cells using the PureLink Genomic DNA Extraction Mini Kit (Invitrogen) according to the manufacturer’s instructions. Five-hundred nanogram of purified genomic DNA was then bisulfite converted using the EpiJET Bisulfite Conversion Kit (Thermo Scientific) according to the manufacturer’s instructions. The rDNA locus was amplified from the bisulfite-converted DNA using primers specific to bisulfite-converted sequences in the human rDNA locus^36^ (GenBank accession code U13369). Primer sequences are as follows: 5′-GTT TTG GGG TTG ATT AGA-3′ and 5′-AAA ACC CAA CCT CTC C-3′. Amplified DNA was cloned into the pCR4-TOPO vector. Individual colonies were isolated and plasmid DNA was purified using a Plasmid DNA Miniprep Kit (Qiagen). Purified DNA was subjected to Sanger sequencing. Clones exhibiting incomplete cytosine conversion were discarded; completely converted clones were analyzed to determine the proportion of methylated to unmethylated CpGs.

### Quantitative PCR (qPCR) of ribosomal RNAs

For quantitative analysis of 28 S and 18 S ribosomal RNA abundance, total RNA was prepared from fibroblasts using the RNeasy RNA Mini Kit (Qiagen) according to the manufacturer’s instructions. Reverse transcription was performed on 1 ug of purified total RNA to generate cDNA using the Quantitect Reverse Transcription Kit (Qiagen) and random hexamer primers according to the manufacturer’s instructions. The cDNA was diluted 1:10 before analysis in qPCR reactions using Sybr Green detection (Sybr Green Master Mix, ABI). qPCR primers for human GAPDH, human 28 S rRNA, and human 18 S rRNA were used; each designed to generate an amplicon of 100–200 bp. qPCR data was analyzed using the ΔΔCt method, normalizing rRNA values to GAPDH values. GAPDH primers: 5′- TGC ACC ACC AAC TGC TTA-3′ and 5′-GGA TGC AGG GAT GAT GTT C-3′. 28 S rRNA primers: 5′- CTA AAT ACC GGC ACG AGA CC-3′ and 5′-TTC ACG CCC TCT TGA ACT CT-3′. 18 S rRNA primers: 5′-GAT GGT AGT CGC CGT GCC-3′ and 5′-GCC TGC TGC CTT CCT TGG-3′.

### Statistical analysis

Data for all non-proteomic analyses were generated from cells pooled from at least two independent experiments containing at least two technical replicates. Distributions are presented as mean ± SEM unless indicated otherwise. In cases where pairwise comparisons of normally distributed data were made, significance was determined by unpaired *t*-test. In cases where parts-of-whole analyses were done, significance was determined by *χ* ^2^-test. Analyses were performed in GraphPad Prism. Cells were randomly selected for analysis, and no samples were excluded. Significant differences are indicated as * for *p* < 0.05; ** for *p* < 0.01; *** for *p* < 0.001; and **** for *p* < 0.0001.M

### Data availability

Proteomics data have been deposited into the ProteomeXchange Consortium via the PRIDE partner repository, accessible at www.ebi.ac.uk/pride. The accession codes are as follows: PXD006012, PXD006013, PXD006014, PXD006015, and PXD006016.

## Electronic supplementary material

Peer Review File

Supplementary Data 1

Supplementary Data 2

Supplementary Data 3

Supplementary Information
