# Supplementary material for: Nucleolar expansion and elevated protein translation in premature aging
Source: Nat Commun. 2017 Aug 30;8:328. doi: 10.1038/s41467-017-00322-z (PMC5577202; doi:10.1038/s41467-017-00322-z)
Supplement: Supplementary file 5 — Supplementary Information [file 41467_2017_322_MOESM5_ESM.pdf]

File Name: Peer Review File
Description:

File Name: Supplementary Information
Description: Supplementary Figures, Supplementary Table.

File Name: Supplementary Data 1
Description: Average %heavy isotope for proteins identified in both WT (AG3258) and HGPS (AG11498) nuclei after 0 days, 2 days, 4 days, and 6 days of labeling with heavy isotope. %heavy isotope was determined by computing the peak area ratio of a heavy-isotope-labeled mass spectrum to the light-isotope-labeled mass spectrum. Ratios were averaged for all peptides identified for each protein.

File Name: Supplementary Data 2
Description: SILAC abundance analysis of equal masses of protein prepared from crude nuclei, cytosol, and chromatin (see Methods). WT cells (AG3258) were completely labeled with heavy isotope while HGPS cells (AG11498) were maintained in light isotope containing media. Proteins identified by more than 3 sequence counts are shown. Peptide ratios were calculated as the peak area ratio of the heavy isotope-containing mass spectrum to the light isotope-containing mass spectrum; ratios were averaged for all peptides identified for each protein. %heavy greater than 50% indicates enrichment in WT cells; %heavy less than 50% indicates enrichment in HGPS cells. Analysis by protein class was performed by searching the dataset for manually curated lists of UniProt IDs. A ribosome biogenesis class was defined as the top 100 hits from the ribosome biogenesis screen described in Badertscher et al Cell Reports 2015.

File Name: Supplementary Data 3
Description: SILAC abundance analysis of equal masses of protein prepared from crude nuclei (see Methods). WT cells (PRF168) were completely labeled with heavy isotope while HGPS cells (PRF167) were maintained in light isotope containing media. Proteins identified by more than 3 sequence counts are shown. Peptide ratios were calculated as the peak area ratio of the heavy isotope-containing mass spectrum to the light isotope-containing mass spectrum; ratios were averaged for all peptides identified for each protein. %heavy greater than 50% indicates enrichment in WT cells; %heavy less than 50% indicates enrichment in HGPS cells. Analysis by protein class was performed by searching the dataset for manually curated lists of UniProt IDs.

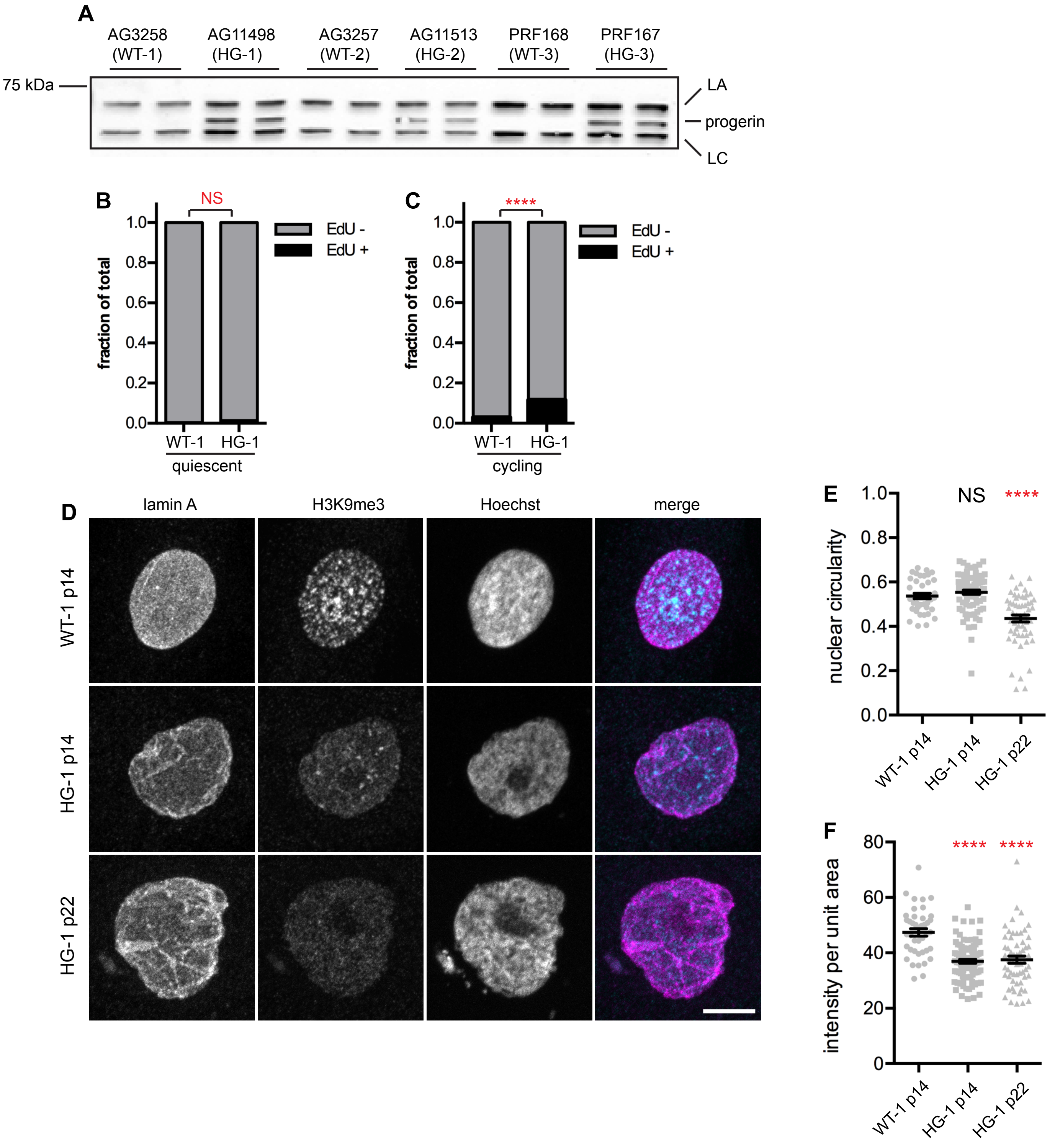

Supplementary Figure 1. (A) Representative Western blot showing expression of lamins A/C in wild type fibroblasts (AG3258, AG3257, PRF168) and expression of lamins A/C and progerin in HGPS fibroblasts (AG11498, AG11513, PRF167). (B) Analysis of quiescence in WT vs. HGPS cells by EdU incorporation for 8 hours in quiescent culture conditions. (C) Analysis of cell cycling rate in WT vs. HGPS cells by EdU incorporation for 4 hours in cycling culture conditions. (B,C) N>297 cells per condition from 4 technical replicates; significance determined by $\chi 2$ test. (D-F) Analysis of nuclear circularity and H3K9me3 levels in WT and HGPS cells. Scale bar, 10 μm. (E) Nuclear circularity becomes significantly distorted only at later passages in HGPS cells. N > 39 cells per condition from 2 technical replicates. (F) H3K9me3 loss is observed at all passages of HGPS cultures tested. N > 41 cells per condition from 2 technical replicates. **** indicates p<0.0001; significance determined by t-test. Bars indicate mean ± SEM.

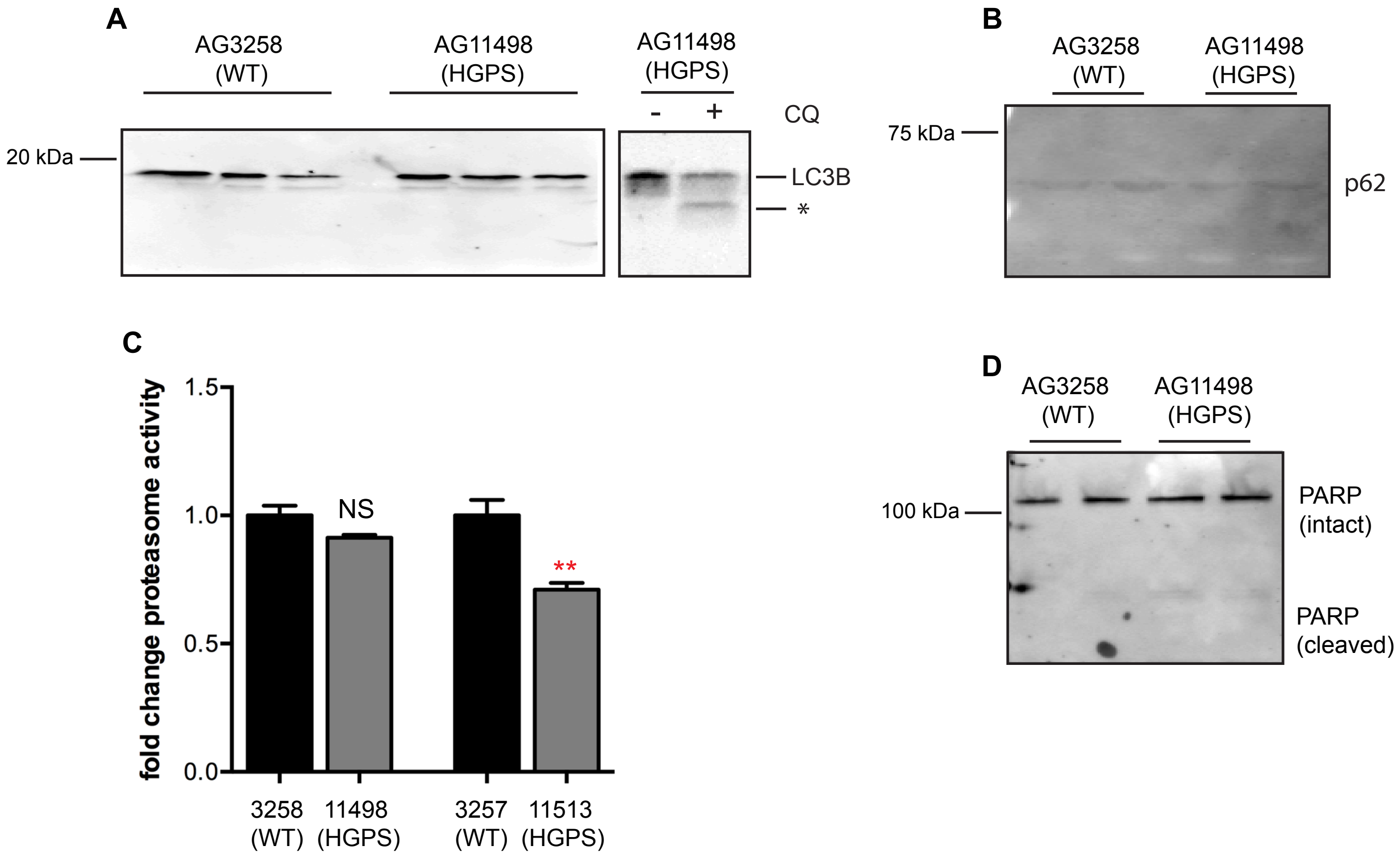

Supplementary Figure 2. (A) Western blot for autophagy proteins LC3B (A) or p62 (B) in quiescent WT and HGPS fibroblasts. Cells were treated for 2 hrs with chloroquine (CQ) to induce accumulation of autophagosomes. Representative data from 1 of 2 replicate experiments shown. (C) Evaluation of proteasome activity by fluorometric tracking of LLVY-AMC cleavage in WT and HGPS fibroblasts. Columns and bars indicate mean ± SEM. ** indicates $p < 0.01$; significance determined by t-test. Representative data from 1 of 2 replicate experiments shown. (D) Western blot for PARP in quiescent WT and HGPS fibroblasts. Representative data from 1 of 2 replicate experiments shown.

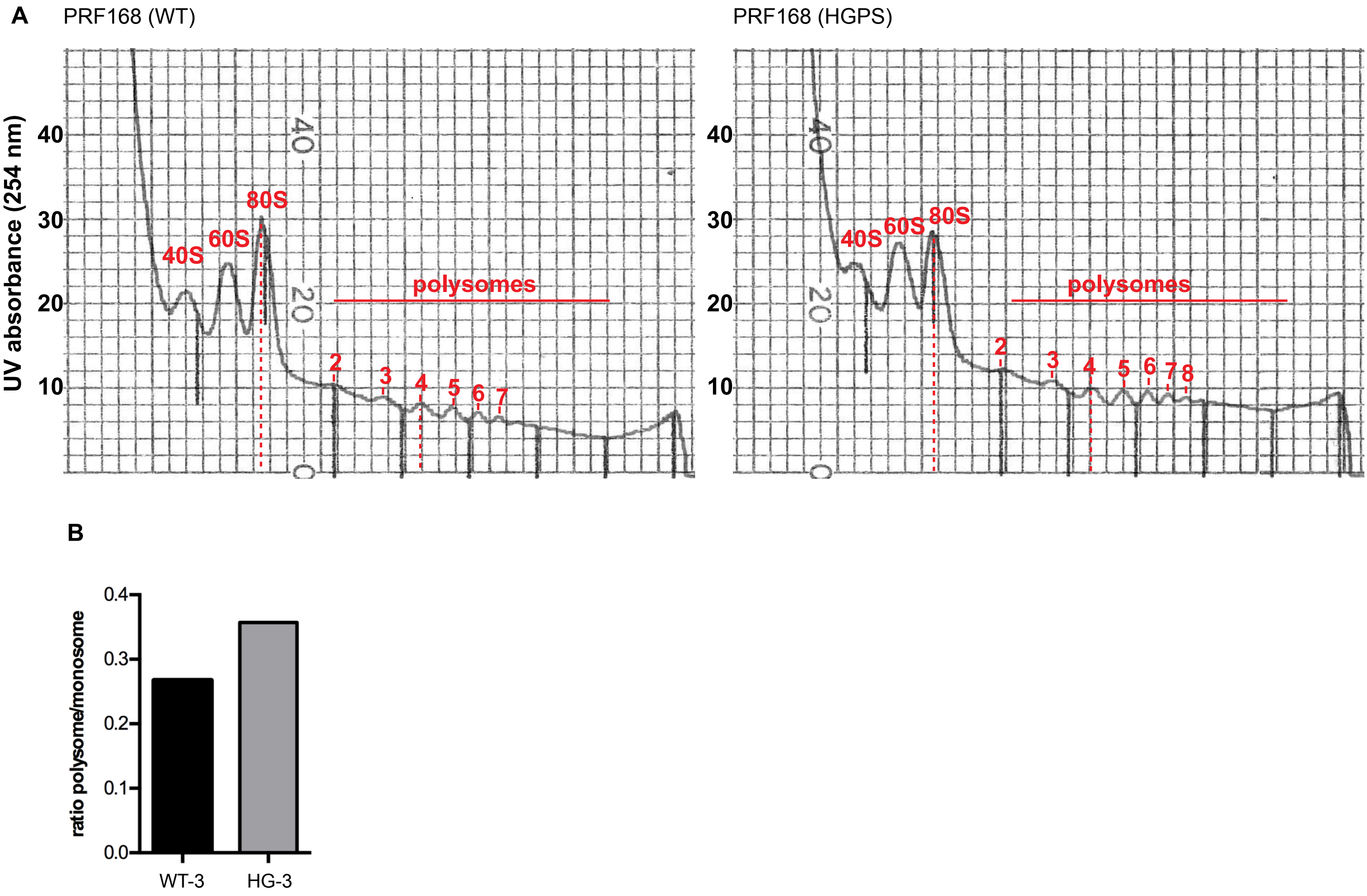

Supplementary Figure 3. Polysome profiles of ribosome populations in WT vs HGPS cells. (A) Polysome profiles. (B) Ratio of monosome to ribosome was determined by calculating the ratio of heights of the monosome (80S) peak to the 4-mer polysome peak. Representative data from 1 of 2 replicate experiments shown.

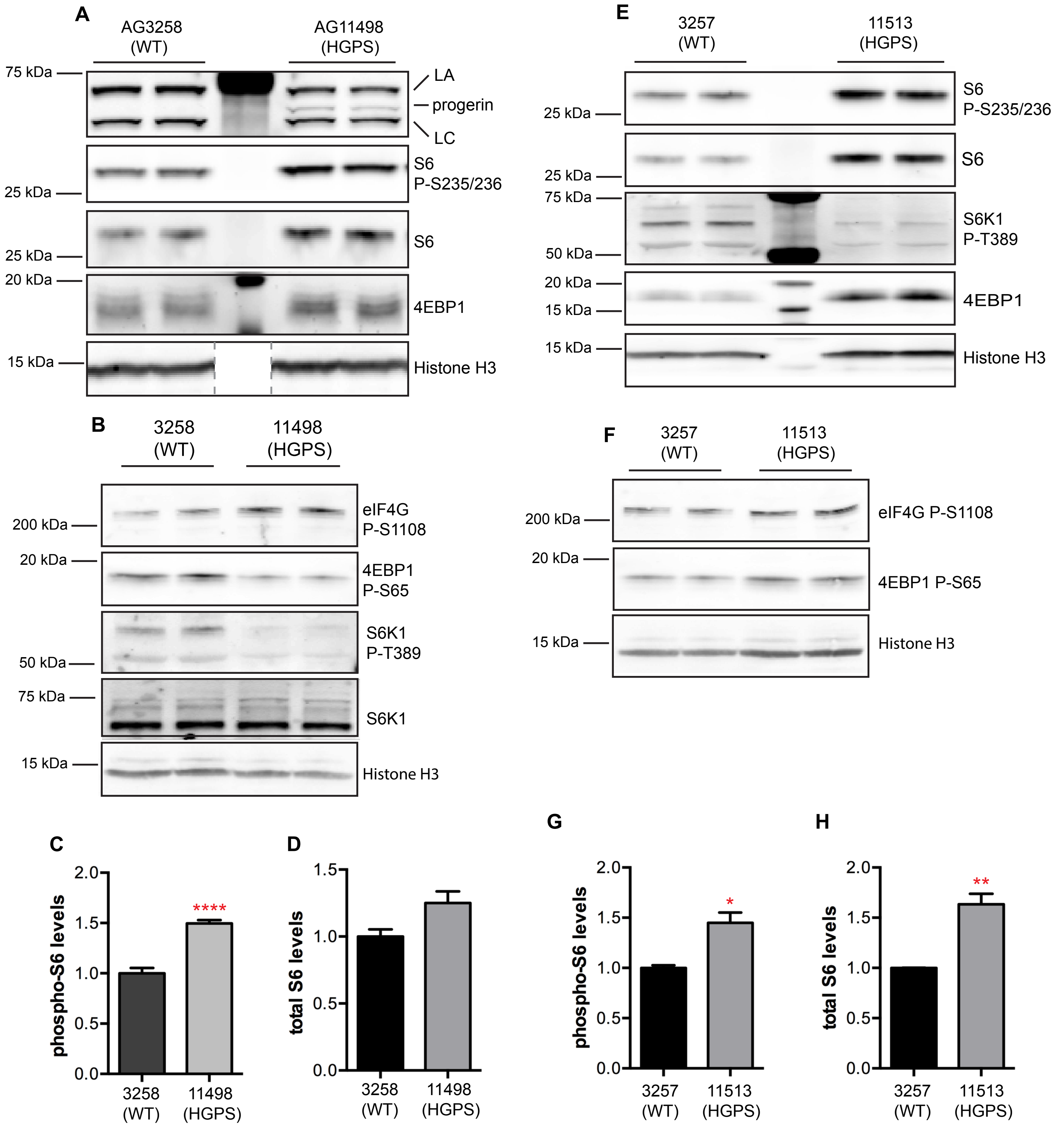

Supplementary Figure 4. Western blot analysis of phosphorylation status of the mTOR substrates ribosomal protein S6 (RPS6), S6K1, eIF4G, and 4EBP1 in WT vs. HGPS cells. Representative data shown from 1 of 2 independent experiments. Paired comparisons were made between AG3258 (WT) and AG11498 (HGPS) (A-D) and AG3257 (WT) and AG11513 (HGPS) (E-H). Columns and bars indicate mean ± SEM, n = 4 from 2 independent experiments. Significance determined by t-test. See also Supplementary Figure 14 for un-cropped Western blot images.

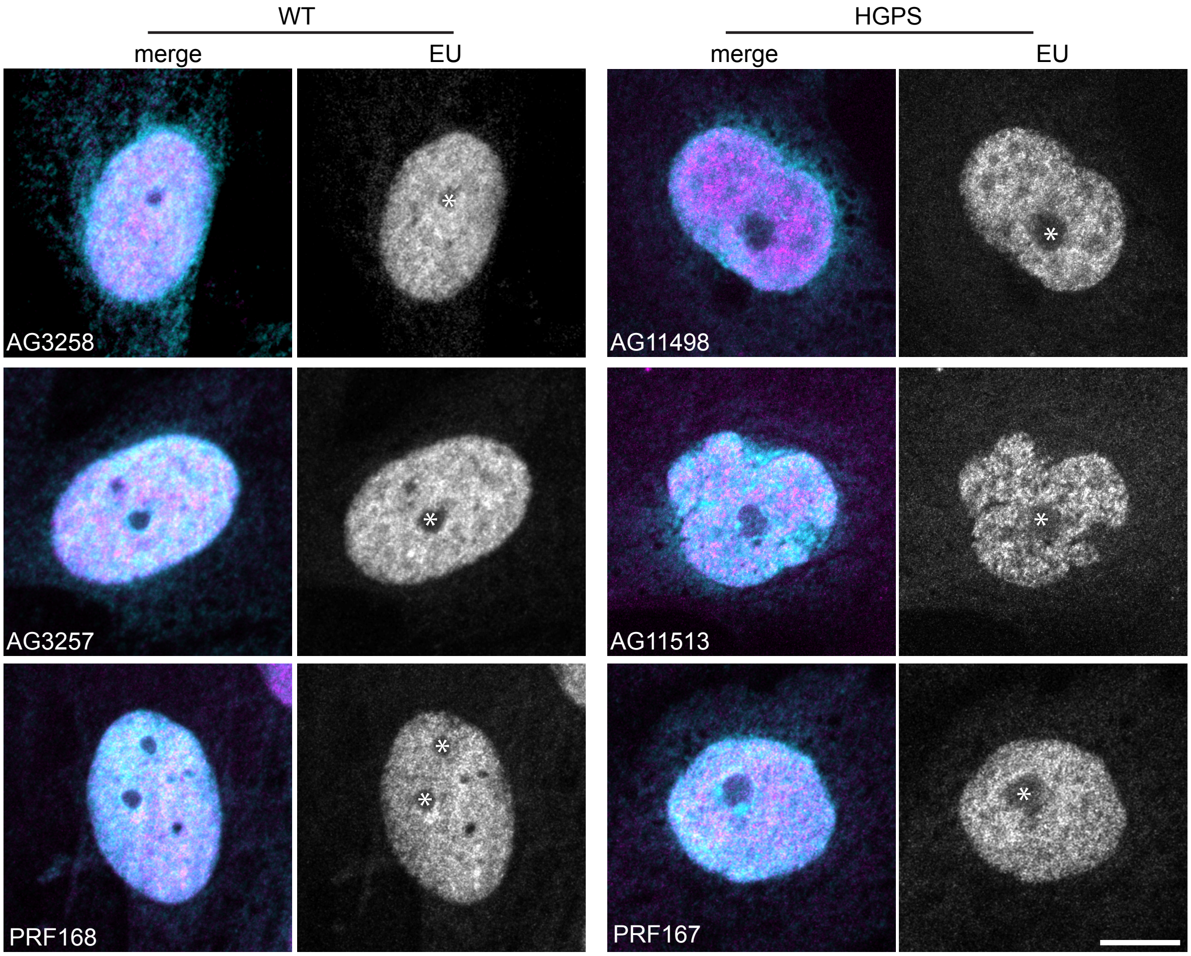

Supplementary Figure 5. Nucleolar RNA labeling is Pol I-specific. Co-incubation of WT and HGPS fibroblasts with EU and 0.1 μg/ml actinomycin D for 4 hr. Nucleoli are marked by asterisks. Representative data from 1 of 2 technical replicates shown. Scale bar, 10 μm.

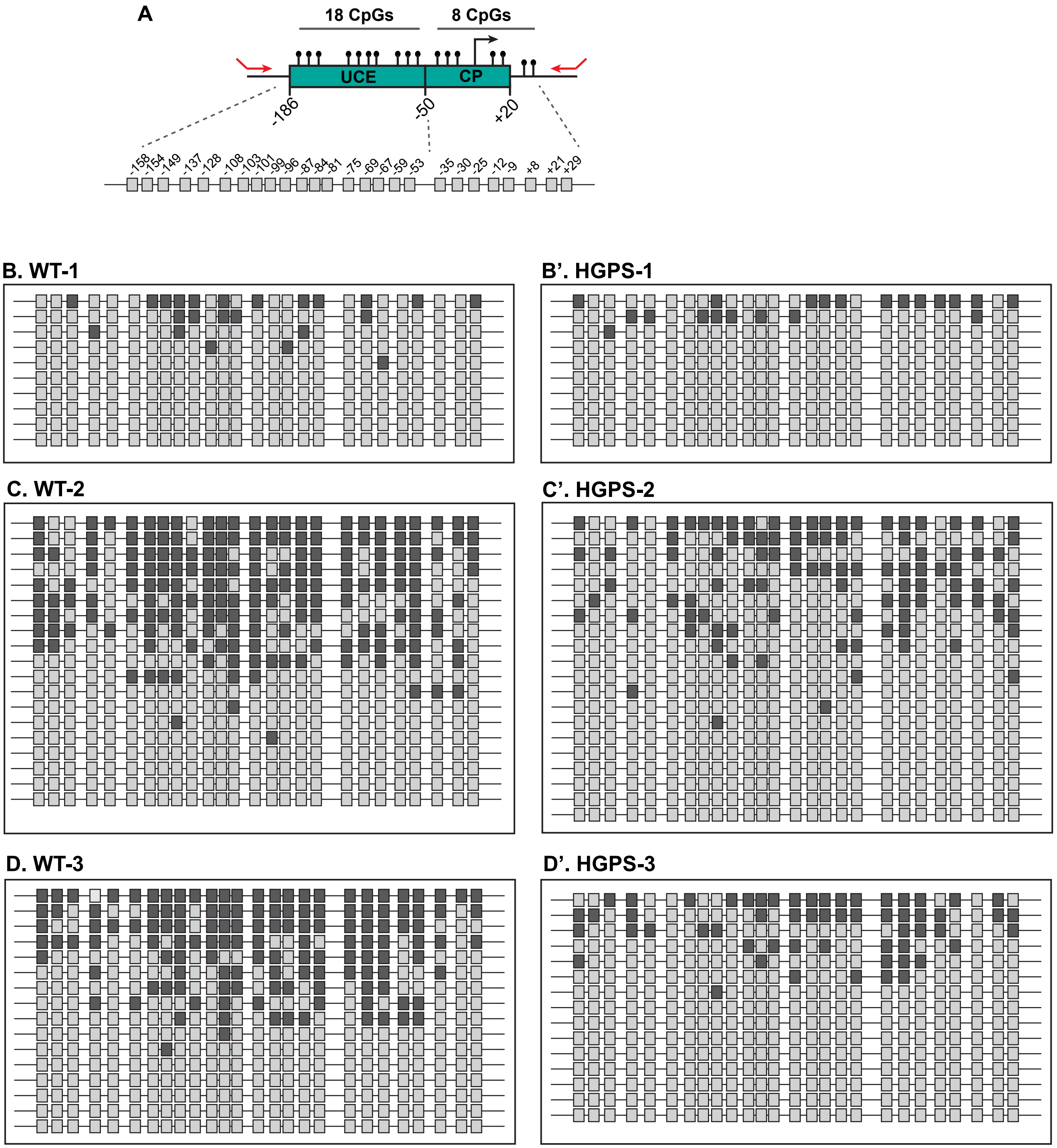

Supplementary Figure 6. Site-by-site bisulfite mapping of CpG methylation at rDNA loci. (A) Schematic representation of the human rDNA promoter, including the upstream control element (UCE, -158 to -53) and core promoter (CP, -35 to +29) with 26 CpG methylations analyzed. (B-D') CpG methylation in 3 pairs of WT and HGPS cells. Light gray indicates an un-methylated CpG and dark gray indicates a methylated CpG. Each line represents an individual clone. Data derived from 2 independent experiments.

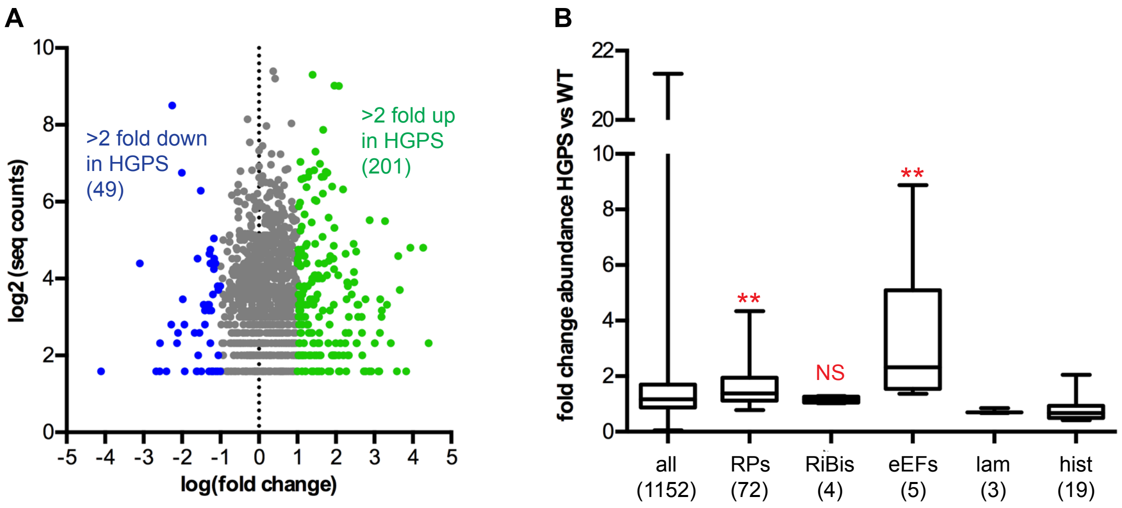

Supplementary Figure 7. Analysis of protein abundance in additional HGPS cell lines. (A) Plot of sequence counts versus fold change in abundance on a logarithmic scale for 1152 proteins identified in nuclei prepared from SILAC-labeled wild type (PRF168) and HGPS (PRF167) cells. (B) Analysis of protein abundance by functional class. RPs, ribosomal proteins; RiBis, ribosome biogenesis proteins; eEFs, translation elongation factors; eIFs, translation initiation factors; lam, lamins; hist, histones; UPR, UPR target genes. Boxes indicate 25th-75th percentile, lines indicate median value, and bars indicate range of values. Significance determined by Mann-Whitney U Test.

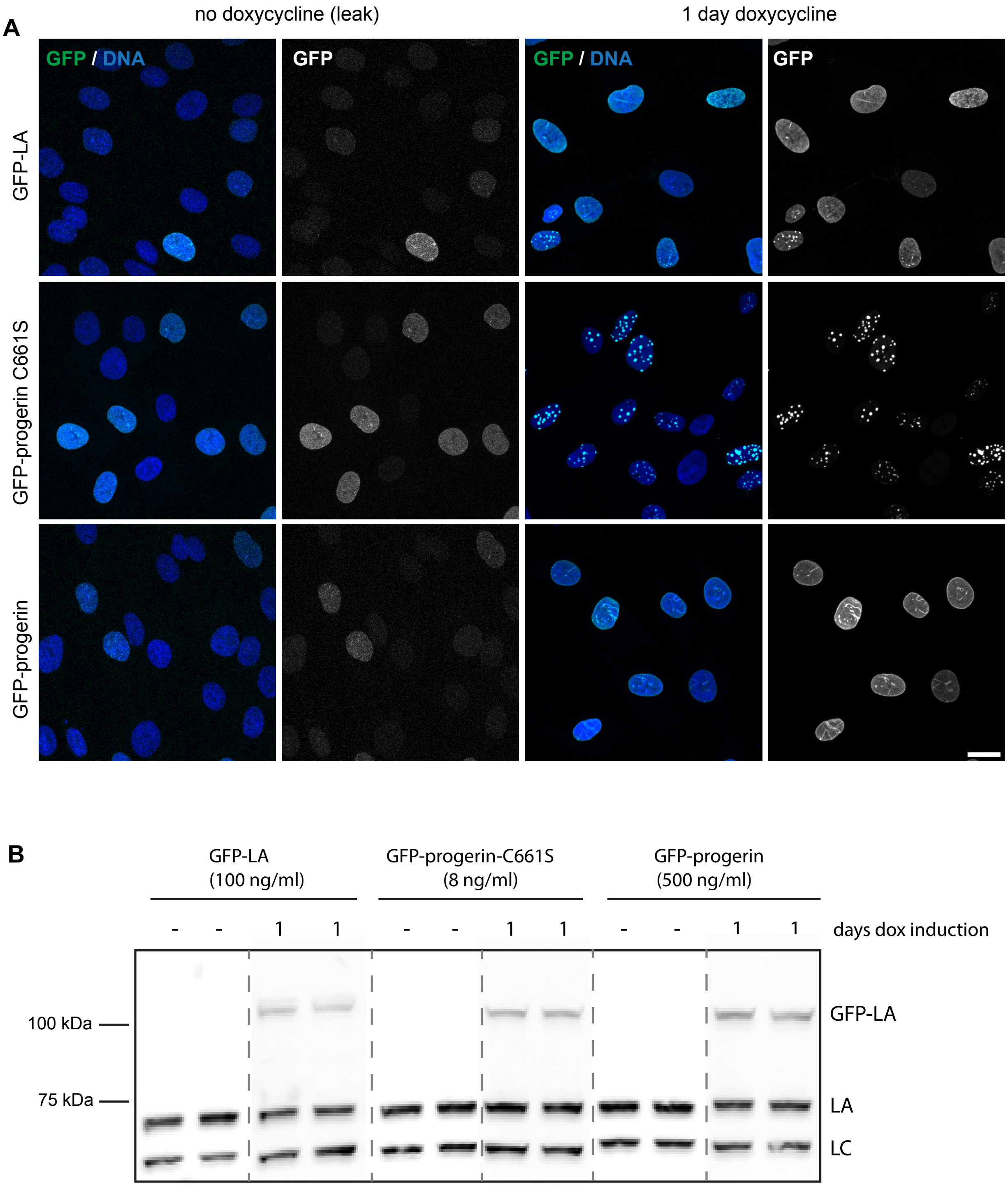

Supplementary Figure 8. (A) Fluorescent detection of GFP-lamin A, GFP-progerin, and GFP-progerin C661S inducible expression in human fibroblasts without doxycycline (A, left panel) and after 1 day doxycycline treatment (A, right panel). Scale bar, 20 μm. (B) detection of GFP fusion protein expression by Western blotting. Doxycycline levels were titrated to allow for roughly equivalent induction of each GFP fusion protein.

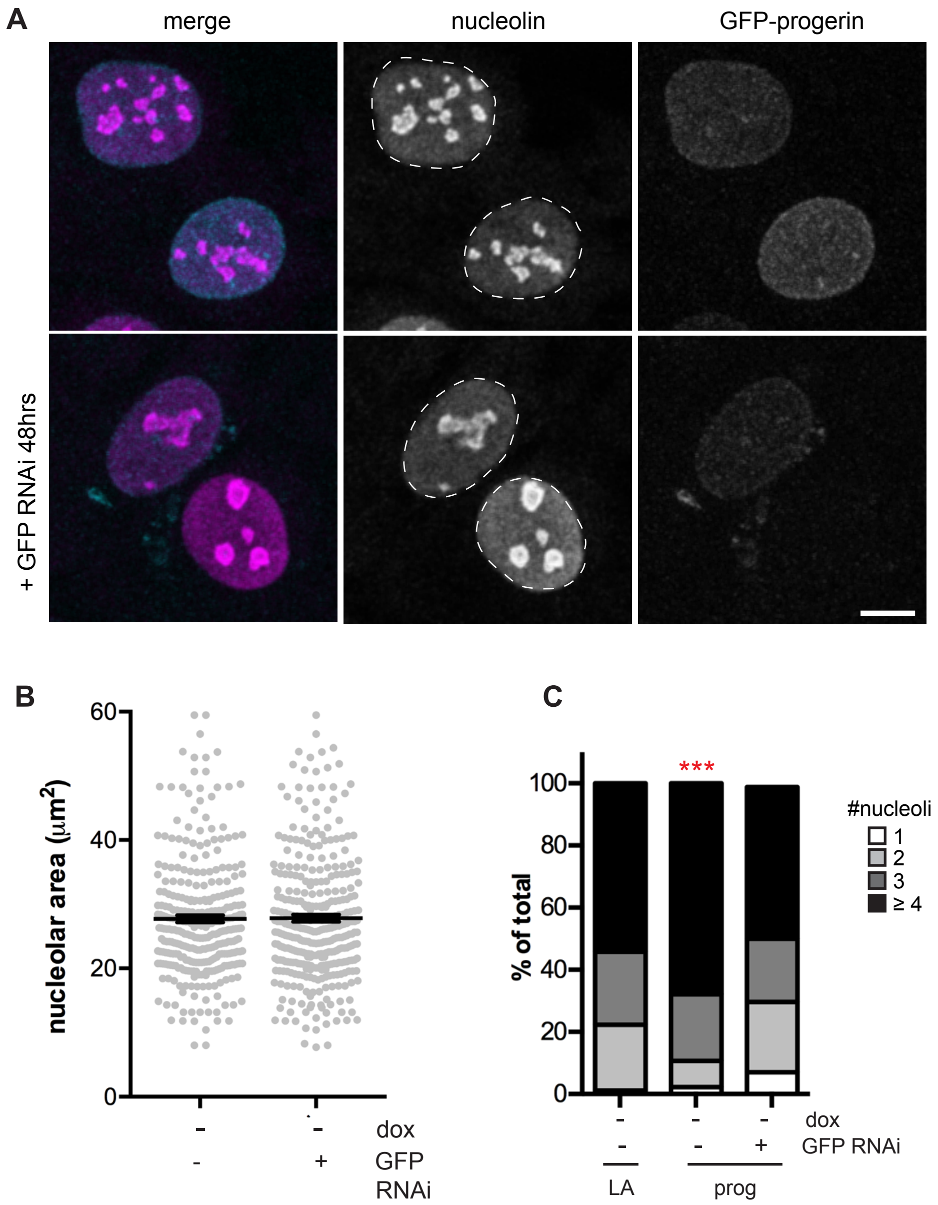

Supplementary Figure 9. anti-GFP RNAi diminishes leaky GFP-progerin expression and nucleolar fragmentation. GFP RNAi diminishes "leak" expression of GFP-progerin (A) and ameliorates nucleolar fragmentation associated with GFP-progerin expression (C) but does not diminish nucleolar size (B). (B,C) N > 130 cells per condition from 2 independent experiments with 2 technical replicates. Bars indicate mean ± SEM. Scale bar,10 μm.

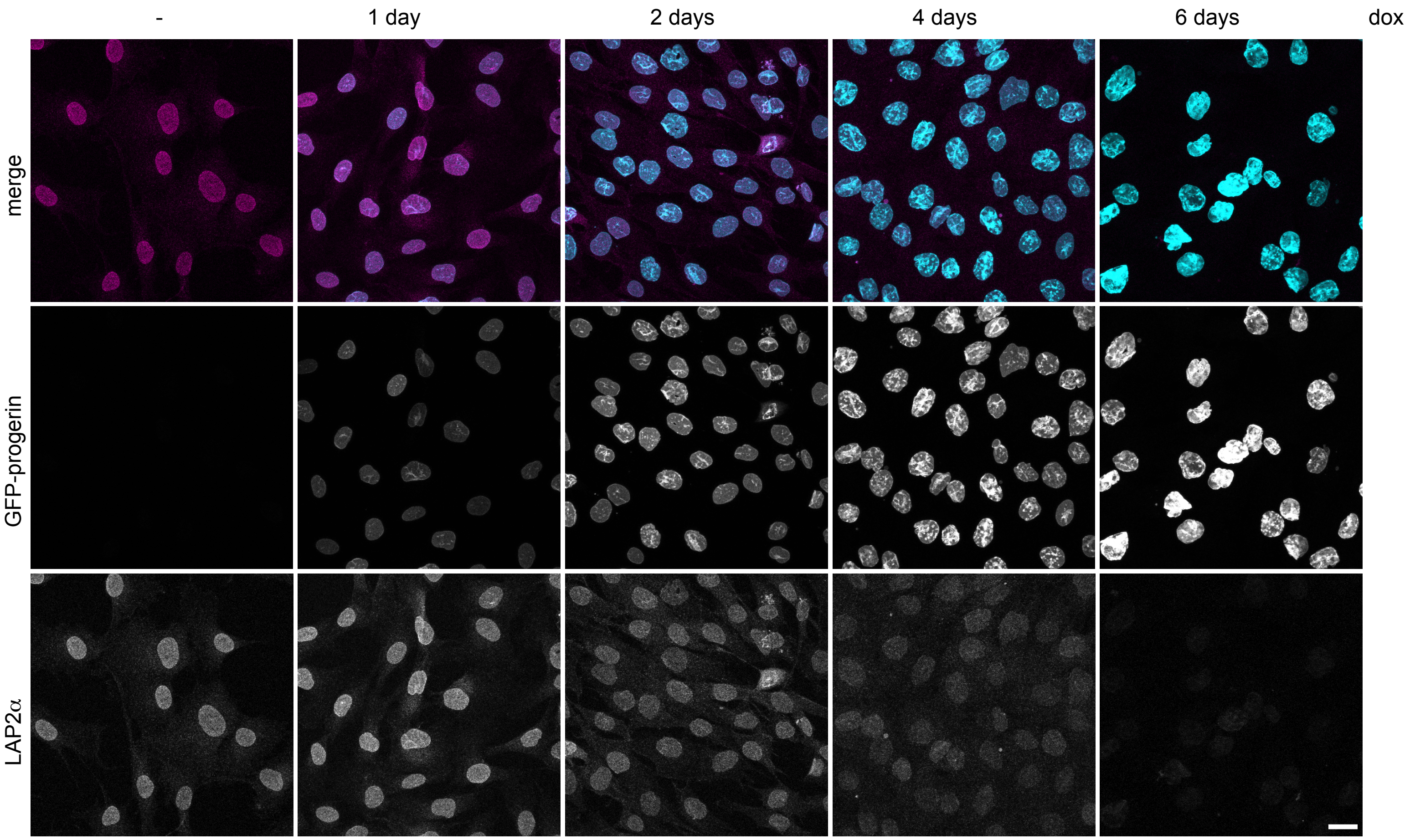

Supplementary Figure 10. Progerin induction and LAP2α loss. Time course of GFP-progerin (cyan) induction by doxycycline and concomitant decrease of LAP2α (magenta). Scale bar, 20 um.

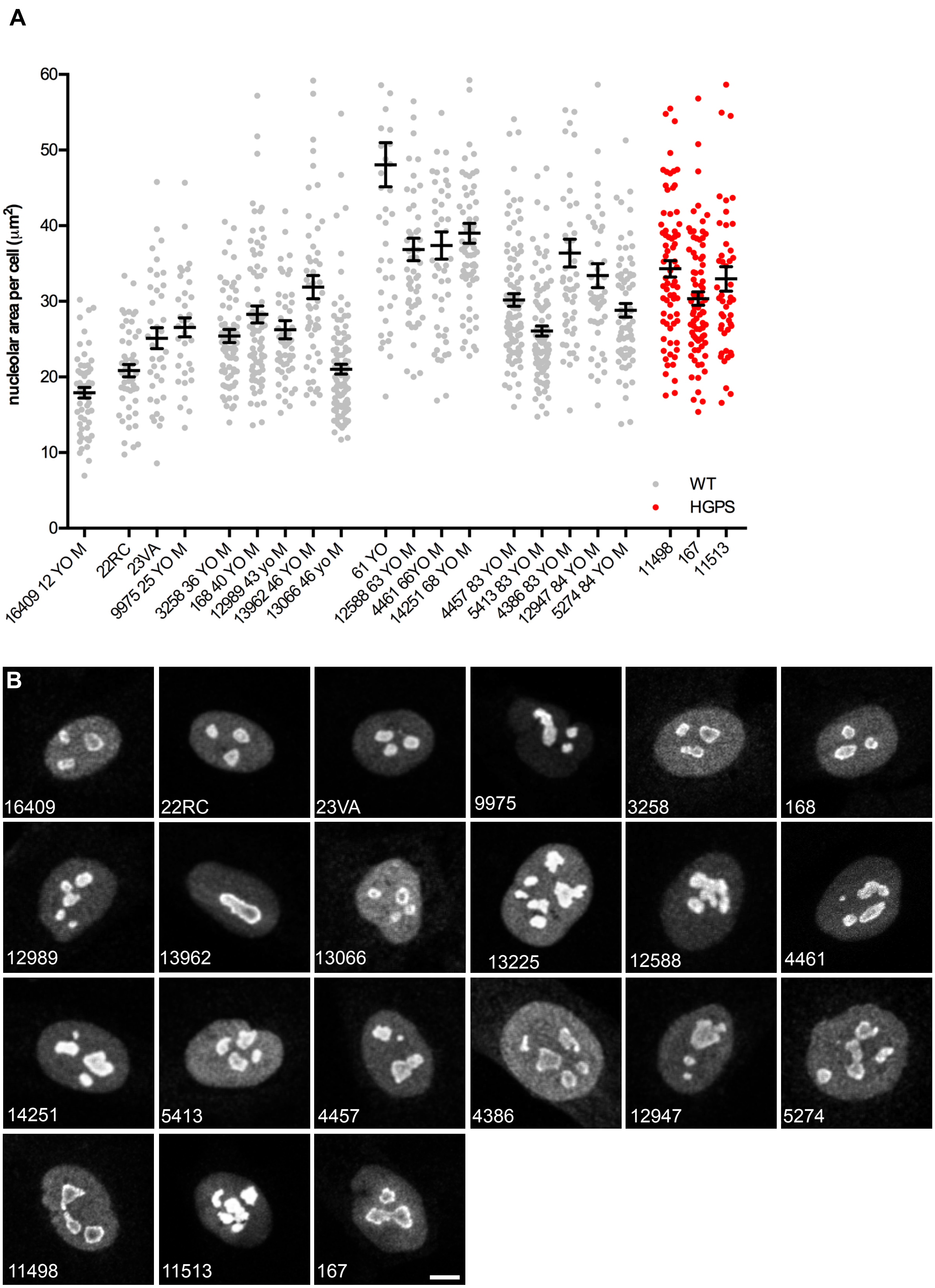

Supplementary Figure 11. Nucleolar size versus age in primary human fibroblasts. (A) Total nucleolar area per cell, determined by boundaries of nucleolin immunofluorescence. Bars indicate mean ± SEM for n > 34 cells per condition from 1-2 independent experiments with 2 technical replicates. (B) Representative nucleolin immunofluorescence. Scale bar, 10 μm. See also Supplementary Table 1.

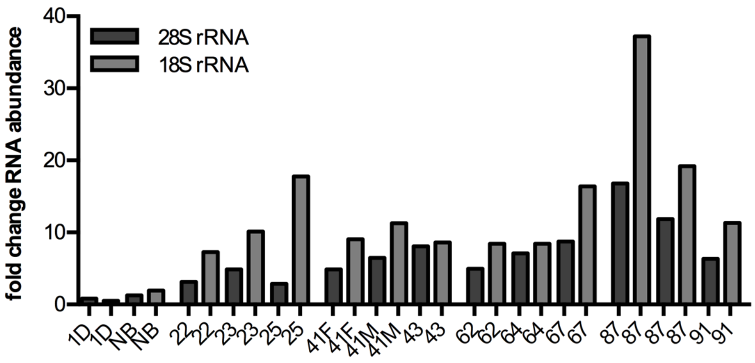

Supplementary Figure 12. qPCR analysis of rRNA levels across age in primary human fibroblasts. 28S and 18S rRNA levels, normalized to GAPDH in the indicated samples. Representative data from 1 of 2 independent experiments shown. See also Supplementary Table 2.

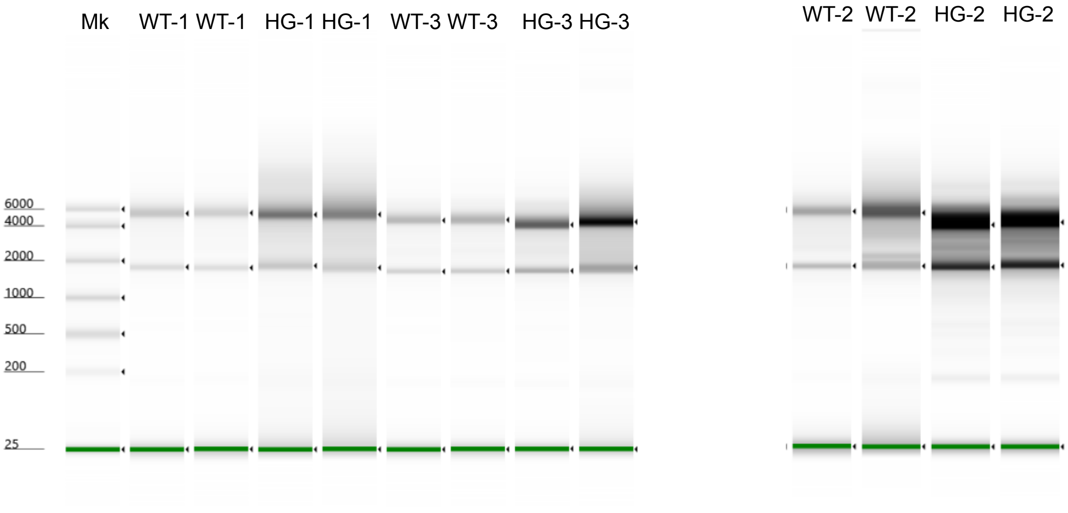

Supplementary Figure 13. Raw images related to Figure 3. Electrophoresis of total cellular RNA in the indicated WT or HGPS cells.

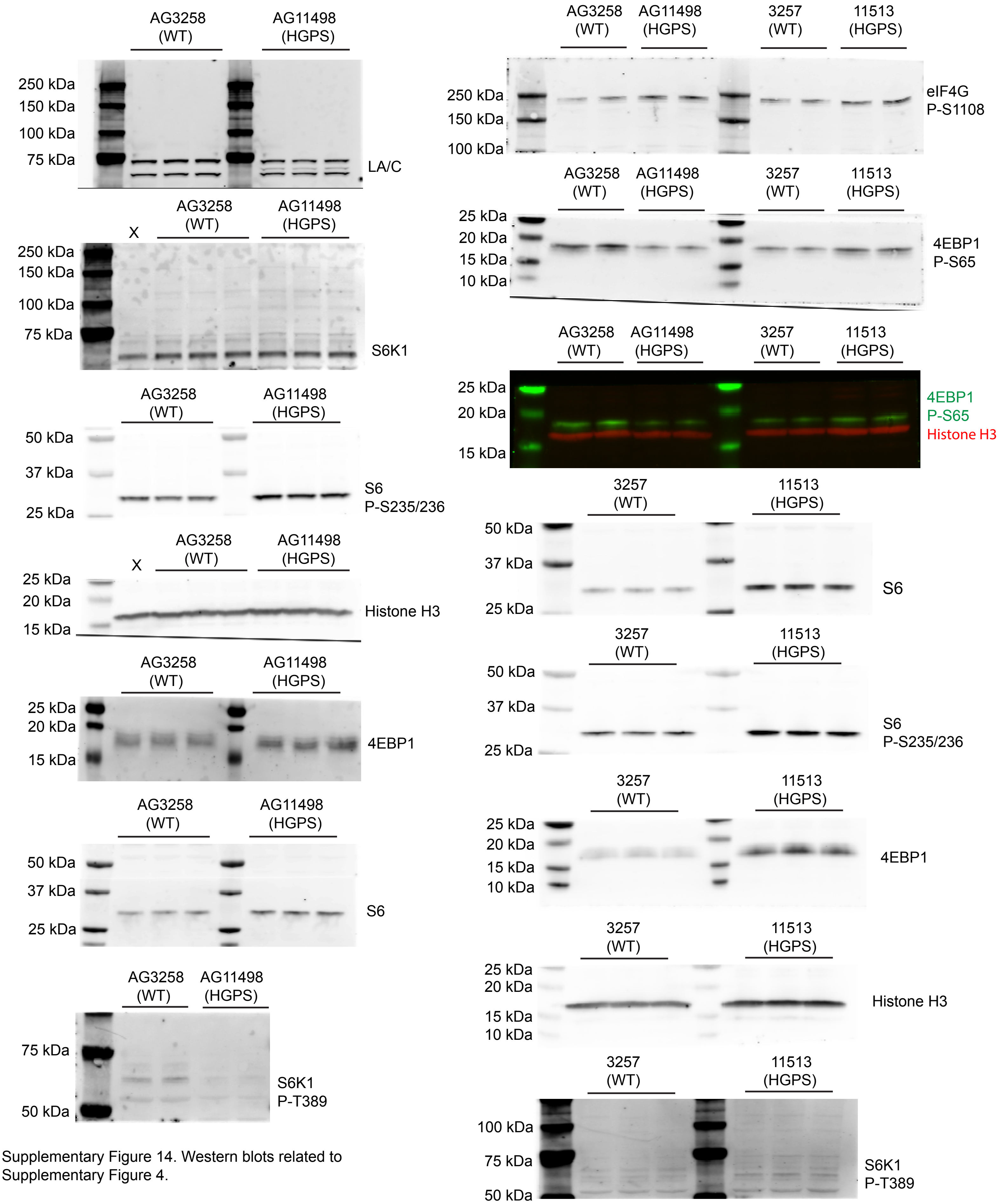

Supplementary Figure 14. Western blots related to Supplementary Figure 4.

Supplementary Table 1.

cell line ID	source	sex	age	genotype
AG16409	Coriell	M	12	WT
22RC	biopsy	M	22	WT
23YA	biopsy	M	23	WT
AG09975	Coriell	M	25	WT
AG3258	Coriell	M	36	WT
PRF168	PRF	M	40	WT
AG12989	Coriell	M	43	WT
AG13962	Coriell	M	46	WT
AG13066	Coriell	M	46	WT
AG13225	Coriell	M	61	WT
AG12588	Coriell	M	63	WT
AG04461	Coriell	M	66	WT
AG14251	Coriell	M	68	WT
AG04386	Coriell	M	83	WT
AG04457	Coriell	M	83	WT
AG05413	Coriell	M	83	WT
AG12947	Coriell	M	84	WT
AG05274	Coriell	M	84	WT
AG11498	Coriell	M	14	HGPS
AG11513	Coriell	F	8	HGPS
PRF167	PRF	M	8	HGPS

List of primary human fibroblasts analyzed by immunofluorescence.

Supplementary Table 2.

cell line ID	source	sex	age	genotype
AG22153	Coriell	M	1 day old	WT
AG21753	Coriell	M	1 day old	WT
22RC	biopsy	M	22	WT
23YA	biopsy	M	23	WT
25MH	biopsy	F	25	WT
AG11745	Coriell	F	41	WT
AG13244	Coriell	M	41	WT
AG12493	Coriell	F	43	WT
62MP	biopsy	M	62	WT
64JW	biopsy	F	64	WT
67LR	biopsy	F	67	WT
AG05247	Coriell	F	87	WT
AG10884	Coriell	M	87	WT
AG07725	Coriell	M	91	WT

List of primary human fibroblasts analyzed by qPCR.
