# Supplementary material for: Nucleolar expansion and elevated protein translation in premature aging
Source: Nat Commun. 2017 Aug 30;8:328. doi: 10.1038/s41467-017-00322-z (PMC5577202; doi:10.1038/s41467-017-00322-z)
Supplement: Supplementary file 1 — Peer Review File [file 41467_2017_322_MOESM1_ESM.pdf]

Reviewers' Comments:

Reviewer #1 (Remarks to the Author)

Buchwalter and Hetzer report that quiescent fibroblasts from Hutchinson-Gilford progeria syndrome (HGPS) patients show increased protein turnover. They also report their failure to find an increase in autophagy and even a moderate reduction in proteasomal protein degradation. By contrast, they report a dramatic increase in protein synthesis rate in HGPS cell lines. They conclude that increased protein turnover in HGPS cells is due to elevated protein synthesis and not to increased degradation. Next, the authors have analyzed mTOR signaling in these cells and found that this pathway is not hyperactivated. By contrast, they observe higher levels of the ribosomal protein RPS6, enlarged nucleoli, increased transcription of rRNA, increased rRNA levels and hypomethylation of rDNA loci in 2 out of 3 HGPS cell lines. To further evaluate ribosome biogenesis in HGPS, the authors carried out a proteomic analysis of nuclei from control and progeroid fibroblasts, finding in the later an increase in ribosomal proteins and translation elongation factors. Their results from ectopic expression of GFP-progerin or silencing of lamin A in cultured normal fibroblasts suggest a role of lamin A in regulating nucleoli and support a causative role of progerin in nucleolar abnormalities. Finally, the authors also observe an increase with age in nucleolar size and fragmentation and in levels of rRNA in cultured fibroblasts obtained from healthy donors.

General comment
This manuscript describes some potentially interesting results. Unfortunately, the work is based exclusively on the use of cultured cells and does not provide any mechanistic information that explains how progerin accumulation or lamin A deficiency induces nucleolar expansion and ribosome biogenesis. In the absence of such in vivo data and mechanistic details, this manuscript is too descriptive and preliminary.

Specific points
1. The authors conclude that HGPS cells present increased protein turnover due to enhanced protein synthesis without increased degradation. It is not obvious how this can be possible. Shouldn’t an increased synthesis rate, in the absence of increased degradation, cause a continuous growth in protein content? Indeed, in Figure S2b, although a bad quality blot for p62, it does seem like p62 is reduced in HGPS cells (indicative of increased autophagy). Moreover, an increment in cleaved PARP is also slightly appreciated in Figure S2D. To avoid misinterpretations, western-blots in Figure S2A, B and D should be accompanied by a control of charge by H3. Anyway, if synthesis rate is continuously increased without an apparent continuous growth in protein content, a cellular mechanism to eliminate this excess of protein must be activated and this should be addressed or discussed.
2. All experiments are performed using 3 different HGPS and WT cell lines. However, it is not clear why the conclusion on the increase in polysomes is based solely on the ratio calculated for one cell line. It should be assessed in the other HGPS cell lines available. Also, increase in 28S and 18S RNA in HGPS cells (Figure 3C) would be more accurate if assessed as in figure S10D, by qPCR and relative to a control.
3. They conclude that phosphorylation of 4EBP1 and eIF4G is unchanged in HGPS. However, in Figure S4 it seems quite clear that the ratio of phospho-4EBP1/total 4EBP1 is reduced, which is consistent with the marked reduction in phosphorylated S6K1 shown in that figure.
4. The authors show that S6 kinase phosphorylation is decreased, ruling out an hyperactivation of mTOR in their experimental system. These data seem to be in conflict with the report by Ramos et al (Sci Transl Med 4, 144ra103, 2012), according to which mTOR signaling is elevated in vivo in Lamin A/C-deficient mice. This work should be cited and this remarkable discrepancy needs to be explained.
5. The reduced methylation of rDNA loci and the subsequently increased synthesis of rRNA in HGPS cells also contrasts with the opposite phenomenon reported in an in vivo progeria mouse

model (Osorio et al., Aging Cell 9(6):947-957, 2010). Again, this should be discussed. This discrepancy, along with the previous point, could be due to a different behavior of HGPS cells in vitro or in a physiological context. If this proved to be the case, the relevance of the findings reported in the present manuscript, based solely on the use of cultured cells, would be very limited.
6. The proteomic study performed by the authors to evaluate ribosome biogenesis is based on the use of nuclear protein extracts. Since most of the protein synthesis takes place in the cytoplasm, the reason for studying only the nuclear levels of these proteins is not clear.
7. The authors report an increase with age in nucleolar area and rRNA abundance in fibroblasts from healthy human donors. Are these changes accompanied of more active protein synthesis?

Minor point
1. Is Figure 3A correctly labeled? All the cells shown in that panel are labeled as wt.
2. In figure S4, phosphorylation marks and their total protein should be addressed in the same immunoblot, it is confusing why it is all in separate panels. It is less accurate, difficult to analyze and results can be less reproducible.

Reviewer #2 (Remarks to the Author)

Buchwalter and Hetzer make a number of important observations related to protein translation and nucleolar size in HGPS cells. These findings suggest that loss of intranuclear A-type lamins leads to enhanced nucleolar size and ultimately elevated protein translation levels. Given connections between translation and aging in a range of organisms, it is interesting to speculate that HGPS fit at one extreme of a spectrum regarding these phenotypes. The findings are of high potential impact, but there are a number of issues that need to be addressed.
1. Is it clear that the arrest state of the normal and HGPS cells are identical? Do they arrest at the same position in the cell cycle and to an equal extent upon quiescence? Do HGPS cells senescence upon quiescence or adopt a senescent like-state. A number of controls need to be provided to show that the population is identical with regard to cell cycle position and response to quiescence.
2. Supplemental Figure 1 is a bit difficult to understand. Why are HGPS cells at different passage compared to undefined controls? A more complete legend could be helpful here at the least.
3. Is the number of nucleoli changed in Figure 2C
4. Are the number of rDNA repeats altered in the context of progerin expression?
5. How is Figure 3C normalized?
6. Why are many of the phenotypes only seen in 2 out of 3 HGPS fibroblasts? This should be discussed at least. Are there other differences between the fibroblasts that relate to disease severity or progerin expression etc.?
7. It seems like Dox increases nucleolar area but not number in Figure 5. Does progerin expression affect both but at different levels, or only area?
8. Under at least some fixation techniques, lamin A/C has been reported to cluster around nucleoli in primary cells. This could be highly relevant to the ribosomal phenotypes and should be cited (PMID 11090133)
9. It is interesting that reduced expression of MYC leads to both reduced ribosome biogenesis and enhanced longevity in mice. The authors should consider testing MYC expression and regulation of targets, and comment on links between their findings and the MYC study (PMID 25619689).

Reviewer #3 (Remarks to the Author)

The authors are addressing various aspects of nucleolar structure and function in the context of premature aging. They are mainly using progerin cells expressing a disease form of lamin A, concluding they have larger nucleoli and enhanced ribosome biogenesis (increased rRNA and ribosomal proteins synthesis). The authors propose that the increased protein synthesis detected

in progeria patient cells may lead to premature aging because of energy exhaustion. This is a simple yet efficient proposal.

The nucleolar structure in cells overexpressing progerin, but not a point mutant that cannot be lipid-modified, is profoundly affected showing severe signs of disruption. The specificity of this observation (not seen with point mutant) in the context of human disease (progerin) is significant.

General comments:
-The concept of nucleolar ‘expansion’ (throughout the manuscript, including in the title) is really misleading. By using ‘expansion’, the authors refer to both the nucleolar activity and its morphology. Activity: the authors mean enhanced/increased activity. Morphology: the authors would like to express an increase in size (but should consider structural alterations).

The problem is that it’s not really clear if it’s an increase in nucleolar size they are looking at, or a loss of nucleolar structural integrity (more likely), making a more ‘diffuse’ ('less compact') nucleolar structure to expand in the nuclear space. In fact, the authors themselves refer on lane 156 to ‘more fragmented’ nucleoli. Close inspection of their images convinced me that nucleolar structure is disrupted (sometimes severely).

The increase in activity should be confirmed by other means than the EU labeling (Fig 3). Typically, a classical transcriptional run-on, or a pulse-chase labeling analyzed by denaturing agarose gel should be performed.

More generally, it would be necessary to strengthen the effects observed on rRNA synthesis. The bioanalyzer captures shown in Fig 3c are a good indication, but a detailed pre-rRNA processing analysis by quantitative Northern-blotting would make the paper a better case.

The increase in nucleolar size, or more likely, the loss of intact nucleolar structure (see above) should be addressed more thoroughly. A good start would be to use systematically more than a single nucleolar marker to detect the nucleolus. Typically, using other markers from other subnucleolar compartments would be very useful.

Detailed comments:
-Fig 2c, the authors claim that the HG cells have enlarged nucleoli, but aren’t we simply looking at cells with drastically altered nucleolar structure? This requires further investigation.

- Figure 3, panel A. There is a problem with the labeling of the panels: all six panels are labelled as WT. I assume the bottom series corresponds to the HG samples series.

Fig 3, panel A, showing an increase in rRNA synthesis by means of an EU labelling. This is a core piece of the demonstration. This should be further supported by a transcriptional run-on (dot- or slot-blot analysis), and or a pulse-chase (denaturing agarose gel analysis).

Fig 3, panel C, showing mature rRNA accumulation. This should be complemented by a fully-fledged pre-rRNA processing analysis by Northern-blotting.

Comparison between panels B and C: isn’t it a bit awkward that the HG-2 sample in panel B do not show an increase in EU labeling, while the HG-2 sample in panel C is precisely the one that shows the strongest increase in mature rRNAs?

Similarly, comparison between panels E and panels A and B: isn’t it a bit weird that HG-1 does not show a reduction in rDNA promoter methylation, while it shows a notable increase in EU labeling (panel A), and a moderate increase in mature rRNAs (panel C)?

-Figure 4, SILAC analysis showing an increase in most of the ribosomal proteins. This is a

convincing data. Could you please check in your dataset the abundance of the pre-rRNA processing factors? (a provisional list of >250 factors has been provided in a systematic screen of > 625 nucleolar proteins). Are they also overexpressed? In principle they should be (or at least some of them)?

- Fig 5, second part: the authors finally conclude that the nuclear lamina is required for nucleolar structure (Fig 5, panels d-h). While this is an interesting idea, of course, this data is totally preliminary. Concluding this would require to conduct a more comprehensive study: including the use of additional nucleolar markers (see comment above on nucleolar subcompartments) to assess how they redistribute in cells depleted of lamins, as well as functional nucleolar parameters (several ribosomal biogenesis readouts to start with).

-Figure S10, when the authors refer to 'nucleolar area per cell', I assume they refer to the 'total nucleolar area'. It would also be very important to know if and how the number of nucleoli per cell vary. Indeed, nucleolar fusion, during which small nucleoli unite into larger ones, is a hallmark of cellular senescence.

Minor,
-In the first part of their Discussion (model paragraph) line 189: the ribosomes produced are not more active, I think what the authors want to say is that more ribosomes are produced (but they are similarly active).

Reviewer #4 (Remarks to the Author)

The manuscript entitled "Nucleolar expansion and elevated protein translation in premature aging" by Buchwalter and Hetzer describes the analysis of cell lines derived from Hutchinson-Gilford progeria syndrome (HGPS) patients and their comparison to wild-type fibroblasts. To this end, they study protein turnover by stable isotope labeling, quantify polysome/monosome ratios, and analyze nucleoli microscopically. From these data, they conclude that HGPS involves ribosome overproduction and therefore increased protein synthesis. According to their model, premature ageing in HPGS patients and normal ageing both involve increased production of ribosomes caused by an impaired nucleoplasmin network.

On the positive side, this paper addresses an interesting question and comes up with a new and intriguing hypothesis. On the negative side, there are several fundamental problems that need to be addressed before the manuscript might become acceptable for publication. The first major problem is that increased protein synthesis without increased protein degradation or cell division is not possible. Therefore, the authors need to carefully reformulate their hypothesis. The second major problem is that the proteomic data acquisition and analysis is not comprehensible and appears to be flawed. The authors therefore need to better describe and probably also to re-analyze the proteomic data. I don't think this will impact their main conclusions. However, the data in its current form is not acceptable.

Major points:

1. The authors conclude that HGPS cells show increased translation without notable changes in protein degradation or cell division. (line 89, "we conclude that increased turnover of the nuclear proteome in HGPS (Fig. 1) is due to elevated synthesis of new protein, and not to increased degradation of old protein"). The problem with this statement is that enhanced protein synthesis without increasing protein degradation or cell division would lead to a continuously increasing protein content per cell. Thus, HGPS cells would never reach a steady state and continuously produce more protein. I cannot imagine how this is be possible: Either the cell division rate or protein degradation (or both) must also increase. The only other possibility that comes to mind is that HGPS cells might secrete more proteins than wt cells.

2. There seems to be a problem in the proteomic data analysis: Table S2 contains uniprot identifiers for the different experiments. However, only the 4D label data contains TREMBL IDS such as “tr|A2A274|A2A274_HUMAN”. It thus appears that the different data sets were searched against different databases or filtered differently.

3. Tables S1: The “#peptides” column. This column does not contain the number of unique peptides identified per protein: It is not possible to identify 889 unique tryptic peptides for myosin 9 (P35579). The numbers shown might be spectral counts instead. However, if these are spectral counts, it is not clear what the quality filter criterion “a minimum of two matching peptides per protein” means.

4. Table S1: It is also strange that some proteins were identified with many peptides in wild-type cells and a much smaller number in HGPS cells (for example, P35579 has 889 peptides at 0D in wt but only 90 in HGPDS. In fact, in the latter dataset the coverage was apparently much lower, since only a single protein was identified with more than 100 peptides.

5. Most of the points above also apply to Table S2

6. There is very little information on how exactly the data was acquired and analyzed. Which resolution, AGS target values, dynamic exclusion settings were used on the Tribrid and the Q-Exactive instrument? How was the HPLC performed (which instrument, gradient, how long)? How was the data calibrated? Is it true that semitryptic peptides were allowed “one tryptic cleavage end per peptide” (which would be problematic)? Also, how exactly were protein computed from peptide ratios?

7. The authors should upload their raw data to a public resource and make it available for reviewers. Without this it is impossible to assess data quality.

8. Fig. 3A: This figure only shows wild-type cell lines. Therefore, it cannot be used to compare rRNA synthesis between HGPS cells and wild-type cells. It is not clear where the data in Fig. 3 B comes from.

9. Line 63 and legend to Fig 1, “pSILAC”: The authors did not use “pSILAC” (as in Schwanhausser et al., Proteomics, 2009 and Selbach et al., Nature, 2008) but dynamic SILAC (as in Doherty et al., JPR, 2009 and Schwanhausser et al., Nature, 2011).

10. The authors used quiescent cells. A potential problem is that in this system, a considerable fraction of amino acids that are incorporated into newly synthesized proteins might be recycled. Importantly, in case aa recycling rates differ between wt and HGPS cells, this would look like differences in protein turnover. Therefore, the authors need to carefully check the relative isotope abundance (RIA) in the aminoacyl tRNA precursor pool for both Lys and Arg. The simplest way of doing this is to look at peptides with missed cleavages.

11. Line 74 “Importantly, Histone H3.1, which is not synthesized in quiescent cells25 incorporated little label in normal and HGPS samples (Fig. 1h), indicating that both cell populations maintained quiescence effectively.” I agree that this result is consistent with the idea that cells are largely quiescent. However, there is some label incorporation, indicating that the cells are not fully quiescent. Moreover, since even a small increase in the fraction of dividing HGPS cells would lead to an increase in the apparent protein “turnover”, this aspect must be checked more thoroughly. For example, the authors could use BrdU labeling to carefully check if there is really no cell division (and, most importantly, no difference).

12. Fig. 4 A is not a volcano plot. A volcano plot displays the fold change (on the x axis) against the significance of a change (on the y axis, typically as negative log p-values). Fig. 4 A shows the number of peptides on the y axis, which has nothing to do with the significance of observed

changes.

13. Legend to Fig. 4: “Deviation from a normal distribution confirmed by D’Agostino and Pearson normality test” (also main text, line 135): SILAC-based log2-fold changes in protein abundance are usually not normally distributed. This is even the case when two untreated samples are compared with each other. Therefore, the statistical test is meaningless.

Reviewer #1 (Expert in HGPS; Remarks to the Author):

1. The authors conclude that HGPS cells present increased protein turnover due to enhanced protein synthesis without increased degradation. It is not obvious how this can be possible. Shouldn't an increased synthesis rate, in the absence of increased degradation, cause a continuous growth in protein content? Indeed, in Figure S2b, although a bad quality blot for p62, it does seem like p62 is reduced in HGPS cells (indicative of increased autophagy). Moreover, an increment in cleaved PARP is also slightly appreciated in Figure S2D. To avoid misinterpretations, western-blots in Figure S2A, B and D should be accompanied by a control of charge by H3. Anyway, if synthesis rate is continuously increased without an apparent continuous growth in protein content, a cellular mechanism to eliminate this excess of protein must be activated and this should be addressed or discussed.

We think it unlikely that a modest upregulation in autophagy or apoptosis would account for the destabilization of the proteome that we observe, or effectively counteract the increase in protein synthesis that we observe. We recognize that this shift in protein synthesis must somehow affect cellular protein content, cellular size, or means to dispose of excess protein. Based on a study by Bridger and Kill (Experimental Gerontology, 2004) describing an early "hyper-proliferative" phase in HGPS fibroblasts followed by premature senescence, and on our own observations, our data suggest that HGPS cells cope with increased protein output by dividing more rapidly, at least at earlier passages. This is strongly supported by an EdU incorporation experiment which indicates that HGPS cells cycle more rapidly; this data is now included in Supplementary Figure 1.

2. All experiments are performed using 3 different HGPS and WT cell lines. However, it is not clear why the conclusion on the increase in polysomes is based solely on the ratio calculated for one cell line. It should be assessed in the other HGPS cell lines available. Also, increase in 28S and 18S RNA in HGPS cells (Figure 3C) would be more accurate if assessed as in figure S10D, by qPCR and relative to a control.

While the increase in rRNA levels as presented on normalized electropherograms is quite striking, we also have performed qPCR analysis of 28S and 18S rRNA levels in one pair of WT and HGPS cells normalized to GAPDH RNA levels (see Reviewer Figure 1A). This analysis also indicates that 28S and 18S rRNA are increased in HGPS cells.

3. They conclude that phosphorylation of 4EBP1 and eIF4G is unchanged in HGPS. However, in Figure S4 it seems quite clear that the ratio of phospho-4EBP1/total 4EBP1 is reduced, which is consistent with the marked reduction in phosphorylated S6K1 shown in that figure.

We agree that the data are suggestive of a decrease of 4EBP phosphorylation in one HGPS line (11498) but not in a second HGPS line (11513). S6K1 phosphorylation is decreased in both HGPS lines tested. From this we conclude that the mTOR pathway is not activated in HGPS cells.

4. The authors show that S6 kinase phosphorylation is decreased, ruling out an hyperactivation of mTOR in their experimental system. These data seem to be in conflict with the report by Ramos et al (Sci Transl Med 4, 144ra103, 2012), according to which mTOR signaling is elevated in vivo in Lamin A/C-deficient mice. This work should be cited and this remarkable discrepancy needs to be explained.

We are aware of the conflicting data on mTOR pathway activation in laminopathy mouse models versus *human disease cell lines. While* Ramos et al *see increased mTOR pathway activation in the lmna -/- mouse model of cardiomyopathy, our data in human HGPS cultures (which are heterozygous for the HGPS-causative mutation) is consistent with work from the Collins lab showing no elevation in basal mTOR signaling in human HGPS cells (Cao et al, Sci Trans Med 2011). The lack of genotype-phenotype agreement between mouse models and human HGPS leads us to rely more heavily on data from primary human cells. We feel that discussion of laminopathy mouse models complicates the current manuscript.*

5. The reduced methylation of rDNA loci and the subsequently increased synthesis of rRNA in HGPS cells also contrasts with the opposite phenomenon reported in an in vivo progeria mouse model (Osorio et al., Aging Cell 9(6):947-957, 2010). Again, this should be discussed. This discrepancy, along with the previous point, could be due to a different behavior of HGPS cells in vitro or in a physiological context. If this proved to be the case, the relevance of the findings reported in the present manuscript, based solely on the use of cultured cells, would be very limited.

The Osorio et al *study was performed in a Zmpste24$^{-/-}$ mouse, which is a quite distinct genetic background from human HGPS. In humans Zmpste24 mutation causes restrictive dermopathy, a perinatal lethal disorder that is more extreme than HGPS. We do not assume that the molecular characteristics of HGPS and RD are the same, or that a mouse model of RD should exhibit the same phenotypes as human cultures derived from HGPS. In regards to rDNA methylation specifically, the high sequence divergence between mouse and human rDNA repeats may suggest the existence of distinct modes of regulation.*

6. The proteomic study performed by the authors to evaluate ribosome biogenesis is based on the use of nuclear protein extracts. Since most of the protein synthesis takes place in the cytoplasm, the reason for studying only the nuclear levels of these proteins is not clear.

While protein synthesis takes place in the cytoplasm, ribosome biogenesis and subunit assembly take place in the nucleus, and ribosomal proteins are found in the nucleus in high abundance.

Since our initial submission, we have analyzed the relative protein abundance in the cytoplasmic fractions, where we also have coverage of ribosomal proteins (presumably those in fully assembled ribosomes) and again observed higher levels of these proteins. This data is now included in Supplemental Table 2 and in Figure 4.

7. The authors report an increase with age in nucleolar area and rRNA abundance in fibroblasts from healthy human donors. Are these changes accompanied of more active protein synthesis?

An increase in protein synthesis is one of several possibilities; it is also possible that increased production of ribosomal subunits might instead induce a ribosomal quality control response or other stress response. A thorough analysis of this issue exceeds the scope of the current study.

Minor point
1. Is Figure 3A correctly labeled? All the cells shown in that panel are labeled as wt.
Thank you for pointing this out; we have corrected this error.
2. In figure S4, phosphorylation marks and their total protein should be addressed in the same immunoblot, it is confusing why it is all in separate panels. It is less accurate, difficult to analyze and results can be less reproducible.

Because of similarity in molecular weights and antibodies derived from the same species this was not possible.

Reviewer #2 (Expert in ageing - overlapping with NCOMMS-16-24756; Remarks to the Author):

1. Is it clear that the arrest state of the normal and HGPS cells are identical? Do they arrest at the same position in the cell cycle and to an equal extent upon quiescence? Do HGPS cells senescence upon quiescence or adopt a senescent like-state. A number of controls need to be provided to show that the population is identical with regard to cell cycle position and response to quiescence.

While quiescent HGPS and WT cells exhibit classic quiescent morphology, and the lack of H3.1 turnover indicates that quiescence is maintained in both cell populations, we also performed a confirmatory EdU labeling experiment in quiescent cells. This experiment indicates that WT and HGPS populations maintain quiescence to an equivalent extent. This data is now included in Supplementary Figure 1.

We have not ourselves analyzed senescence in quiescent HGPS cultures, as the intent of performing our SILAC pulse-labeling experiments on quiescent cells was simply to analyze protein turnover in a non-dividing cell population. While all SILAC experiments presented were performed in non-dividing cells to avoid cell division confounding interpretation of results, all other experiments were performed in cycling cells and indicate increases in nucleolar activity and translation output in HGPS cells.

2. Supplemental Figure 1 is a bit difficult to understand. Why are HGPS cells at different passage compared to undefined controls? A more complete legend could be helpful here at the least.

We have clarified this in the legend. A commonly reported phenotype for HGPS cells in culture is distortion of nuclear shape (see for instance Goldman et al PNAS 2004); however, this phenotype becomes apparent only at later passages (as is quantified in Supplemental Figure 1). We show examples of HGPS cells stained at earlier (passage 14) and intermediate (passage 22) stages to show the progressive distortion of nuclear morphology. Another phenotype commonly described in HGPS cells is loss of

heterochromatin marks, which we also see at a significant level (stained for H3K9me3) at all passages analyzed. The relative timing and severity of these cellular phenotypes is consistent with what has been reported by the Misteli group in recent work (Kubben et al Methods 2016).

3. Is the number of nucleoli changed in Figure 2C

We have now included this analysis. Two out of 3 HGPS lines have significantly fewer (but larger) nucleoli than their WT controls.

4. Are the number of rDNA repeats altered in the context of progerin expression?

Expansion of repetitive DNA is a possibility given the genomic instability that arises from loss of constitutive heterochromatin. However, recent work indicates extensive variation in rDNA copy number across populations (Gibbons et al., Nature Comm 2014). Conclusively answering the question of changes to rDNA copy number may require samples from a larger pool of normal and diseased donors than we currently have access to. We have not yet addressed this question, and think its answer lies outside the scope of this study.

5. How is Figure 3C normalized?

Because rRNA comprises such a large proportion of total cellular RNA, we normalized these samples by total cell numbers (RNA samples prepared from 250,000 cells per well; equal proportion loaded in each lane).

6. Why are many of the phenotypes only seen in 2 out of 3 HGPS fibroblasts? This should be discussed at least. Are there other differences between the fibroblasts that relate to disease severity or progerin expression etc.?

Unfortunately we have no information about disease severity from the patient donors. AG03258 (WT), AG11498 (HGPS), PRF168 (WT) and PRF167 (HGPS) are all male; AG03257 (WT) and AG11513 (HGPS) are female. The key phenotypes of increased protein translation and increased production of ribosomal RNA are observed in all 3 lines.

While nucleoli are enlarged but not to a statistically significant extent in the PRF167 line, the size of nucleoli in that cell line is very similar to the other HGPS lines (the matched WT control, PRF168, has larger nucleoli than the other controls). While a significant decrease in rDNA CpG methylation is not observed in AG11498, its control (AG3258) also has very low methylation levels. When comparing the number of rDNA loci identified with any methylation versus *those completely lacking methylation, it is apparent that all HGPS cells have a larger proportion of completely un-methylated rDNA loci. This comparison is now included in Table 1 and is also apparent by looking at clonal methylation maps in Supplementary Figure 6.*

7. It seems like Dox increases nucleolar area but not number in Figure 5. Does progerin expression affect both but at different levels, or only area?

Expression of GFP-progerin does increase both nucleolar number and area. We have changed how nucleolar numbers are presented to a “parts of whole” analysis in Figure 5d rather than only presenting the average number of nucleoli per condition to make this clearer. Even “leak” levels of GFP-progerin increase nucleolar number; if we block this leak by administering anti-GFP RNAi, nucleolar numbers decrease – see Figure S9.

8. Under at least some fixation techniques, lamin A/C has been reported to cluster around nucleoli in primary cells. This could be highly relevant to the ribosomal phenotypes and should be cited (PMID 11090133)

Thank you for pointing this out! We have now included this citation in our discussion.

9. It is interesting that reduced expression of MYC leads to both reduced ribosome biogenesis and

enhanced longevity in mice. The authors should consider testing MYC expression and regulation of targets, and comment on links between their findings and the MYC study (PMID 25619689).

We think the link between myc, nucleoli, and lifespan is a very intriguing one. In general, we think that global loss of heterochromatin in HGPS (as has been described by the Goldman group and Misteli group) allows de-repression of many loci. Since rDNA loci are so abundant and so heavily transcribed, increased production of rRNA emerges as a prominent effect of depleting heterochromatin in HGPS. It is possible that the MYC locus is similarly de-repressed.

Reviewer #3 (Expert in ribosome biogenesis - overlapping with NCOMMS-16-24756; Remarks to the Author):

1. The concept of nucleolar ‘expansion’ (throughout the manuscript, including in the title) is really misleading. By using ‘expansion’, the authors refer to both the nucleolar activity and its morphology. Activity: the authors mean enhanced/increased activity. Morphology: the authors would like to express an increase in size (but should consider structural alterations).

We have now performed additional analysis of nucleolar subcompartments using fibrillarin to stain the dense fibrillar component and UBF1 to mark fibrillar centers. In general this analysis indicates that the tripartite compartmentalization of nucleoli is preserved in HGPS cells; nucleoli generally are larger but still contain fibrillarin and UBF1 in the expected organization. Importantly, we did not observe segregation of fibrillarin or UBF1 into nucleolar “caps” as is sometimes seen under conditions where RNA Pol I activity is inhibited. From this analysis we conclude that nucleoli are likely to be enlarged but functional rather than disrupted. Representative images of fibrillarin and UBF1 staining in WT and HGPS cells are now included in Figure 2, Figure 5, and Figure 6. From this we think that retaining “nucleolar expansion” in the title and text is appropriate.

2. The problem is that it’s not really clear if it’s an increase in nucleolar size they are looking at, or a loss of nucleolar structural integrity (more likely), making a more ‘diffuse’ ('less compact') nucleolar structure to expand in the nuclear space. In fact, the authors themselves refer on lane 156 to ‘more fragmented’ nucleoli. Close inspection of their images convinced me that nucleolar structure is disrupted (sometimes severely).

Please see above. We now also include quantification of nucleolar numbers / cell in our figures.

3. The increase in activity should be confirmed by other means than the EU labeling (Fig 3). Typically, a classical transcriptional run-on, or a pulse-chase labeling analyzed by denaturing agarose gel should be performed.

Because the nucleolar EU labeling was completely blocked by low-dose actinomycin D administration (Figure S5), we are reasonably confident that the EU labeling detected represents rRNA transcription by Pol I.

4. More generally, it would be necessary to strengthen the effects observed on rRNA synthesis. The bioanalyzer captures shown in Fig 3c are a good indication, but a detailed pre-rRNA processing analysis by quantitative Northern-blotting would make the paper a better case.

The main goal of this report is to share the novel finding that translation is increased in premature aging. We are interested in pursuing the mechanism by which normal and mutant lamins influence nucleolar activity and ribosome biogenesis, but think such mechanistic and temporal analyses will require the development of additional tools and are better suited to a more controlled system where progerin synthesis can be induced and/or lamin A expression can be blocked to monitor acute effects on these processes. We intend to pursue this further in future work.

5. The increase in nucleolar size, or more likely, the loss of intact nucleolar structure (see above) should be addressed more thoroughly. A good start would be to use systematically more than a single nucleolar marker to detect the nucleolus. Typically, using other markers from other subnucleolar compartments would be very useful.

Thank you for pointing this out; we have addressed this with additional antibody stains that are now included in Figure 2, Figure 5, and Figure 6.

6. Comparison between panels B and C: isn't it a bit awkward that the HG-2 sample in panel B do not show an increase in EU labeling, while the HG-2 sample in panel C is precisely the one that shows the strongest increase in mature rRNAs? Similarly, comparison between panels E and panels A and B: isn't it a bit weird that HG-1 does not show a reduction in rDNA promoter methylation, while it shows a notable increase in EU labeling (panel A), and a moderate increase in mature rRNAs (panel C)?

We agree that these data may indicate some differences in how rDNA is transcribed among HGPS cell lines. The key phenotypes of increased protein translation and increased production of ribosomal RNA are observed in all 3 lines. While a significant decrease in rDNA CpG methylation is not observed in AG11498, its related control (AG3258) also has very low methylation levels. When comparing the number of rDNA loci identified with any methylation versus *those completely lacking methylation, it is apparent that all HGPS cells have a larger proportion of completely un-methylated rDNA loci. This comparison is now included in Table 1 and is also apparent by looking at clonal methylation maps in Supplementary Figure 6. It is possible that increased rRNA production can be achieved by increased transcriptional rates (which EU incorporation could detect) and/or by increased proportion of rDNA loci becoming active (which loss of CpG methylation reports). It is possible that some HGPS lines rely more on one mechanism than the other, but the net result is consistent upregulation of rRNA levels and protein translation output.*

8. Figure 4, SILAC analysis showing an increase in most of the ribosomal proteins. This is a convincing data. Could you please check in your dataset the abundance of the pre-rRNA processing factors? (a provisional list of >250 factors has been provided in a systematic screen of > 625 nucleolar proteins). Are they also overexpressed? In principle they should be (or at least some of them)?

Since our initial submission, we have acquired additional data consistent with these findings. First, we analyzed cytosolic fractions from the SILAC abundance analysis experiment. In this dataset, we also find significant upregulation of ribosomal subunits. These data are now included in Supplementary Table 2 and in Figure 4. Secondly, we repeated the SILAC abundance analysis on crude nuclei from an additional pair of WT and HGPS lines (PRF168 and PRF167). This second analysis shows results consistent with the first – significant upregulation of ribosomal proteins. These data are now included in Figure S7 and in Supplementary Table 3.

In regard to the upregulation of ribosome biogenesis proteins, we referred to Badertscher et al Cell Reports 2015 and focused our search on the top 100 hits from that screen. In general, we observed modest coverage of RiBi proteins in crude nuclei (and less coverage in cytosolic fractions). We recovered 14/100 of the top 100 RiBi screen hits identified by Badertscher et al. We noticed that while proteins annotated as involved in the small subunit processome arm of ribosome biogenesis (such as nucleophosmin [2.37-fold], nucleolin [1.32-fold], NOP58 [2.15-fold], NOP56 [1.09-fold], and fibrillarin [2.11-fold]) were modestly upregulated in HGPS cells, RiBi screen hits that had multiple functions were not significantly upregulated (for example, the TRiC chaperone complex, and general splicing factors). We might not expect such proteins with important functions in multiple cellular processes to be affected in HGPS, so we chose not to include those in Figure 4. We only include the core RiBi proteins listed above in Figure 4, but all RiBi proteins detected are listed in Supplementary Table 2. As a group, these RiBi proteins are modestly upregulated; because of the modest effect and the sparse coverage of this group, the difference is not significant. While unfortunate that we did not have better coverage of SSU processome RiBi proteins, we look forward to dissecting the effects of lamin depletion or mutation on nucleolar activity in a more defined system in the future.

9. Fig 5, second part: the authors finally conclude that the nuclear lamina is required for nucleolar structure (Fig 5, panels d-h). While this is an interesting idea, of course, this data is totally preliminary. Concluding this would require to conduct a more comprehensive study: including the use of additional nucleolar markers (see comment above on nucleolar subcompartments) to assess how they redistribute in cells depleted of lamins, as well as functional nucleolar parameters (several ribosomal biogenesis readouts to start with).

In this study we mainly focus on the new finding that translation is increased in HGPS cells. However, we did stain additional nucleolar subcompartments with fibrillarin and UBF1 antibodies. This analysis indicates that lamin A depletion causes nucleoli to enlarge but does not disrupt their normal organization. The lamin A depletion data has been moved to a separate figure (Figure 7), and representative images of fibrillarin and UBF1 immunostains are now included.

A detailed analysis of how lamin A controls the biogenesis of nucleoli is an exciting area we will explore in the future. We will need to generate new tools to study the dynamics of nucleolar proteins, perform ultrastructural studies etc. We hope that the reviewer agrees that this is outside the scope of this report.

10. Figure S10, when the authors refer to 'nucleolar area per cell', I assume they refer to the 'total nucleolar area'. It would also be very important to know if and how the number of nucleoli per cell vary. Indeed, nucleolar fusion, during which small nucleoli unite into larger ones, is a hallmark of cellular senescence.

We analyzed nucleolar numbers per cell in a subset of cells from our cellular aging panel and found no significant changes in the numbers of nucleoli per cell with age (please see Reviewer Figure 1b). It is important to note that all cultures analyzed were at very early passage, and an enlarged, single nucleolus may be linked to replicative senescence in culture but less clearly so to the chronological age of donors of freshly isolated primary cultures.

Minor,
-In the first part of their Discussion (model paragraph) line 189: the ribosomes produced are not more active, I think what the authors want to say is that more ribosomes are produced (but they are similarly active).

We will modify this text.

Reviewer #4 (Expert in proteomics; Remarks to the Author):

1. The authors conclude that HGPS cells show increased translation without notable changes in protein degradation or cell division. (line 89, "we conclude that increased turnover of the nuclear proteome in HGPS (Fig. 1) is due to elevated synthesis of new protein, and not to increased degradation of old protein"). The problem with this statement is that enhanced protein synthesis without increasing protein degradation or cell division would lead to a continuously increasing protein content per cell. Thus, HGPS cells would never reach a steady state and continuously produce more protein. I cannot imagine how this is be possible: Either the cell division rate or protein degradation (or both) must also increase. The only other possibility that comes to mind is that HGPS cells might secrete more proteins than wt cells.

Our data suggest that total protein content (including secreted protein output) is elevated in HGPS. Based on a study by Bridger and Kill (Experimental Gerontology, 2004) describing an early "hyper-proliferative" phase in HGPS fibroblasts followed by premature senescence, and our own observations, we expect that HGPS cells cope with increased protein output by dividing more rapidly, at least at earlier passages (which is when all our analyses were performed). We have performed an EdU incorporation experiment that confirms this. This data is now included in Supplementary Figure 1.

2. There seems to be a problem in the proteomic data analysis: Table S2 contains uniprot identifiers for the different experiments. However, only the 4D label data contains TREMBL IDS such as "tr|A2A274|A2A274_HUMAN". It thus appears that the different data sets were searched against different databases or filtered differently.

Thank you for pointing out this issue. The 4D label datasets were inadvertently searched against a larger UniProt database that contained TREMBL IDs. We have repeated the database search with the reference UniProt database used for analyzing all other datasets, which contains only reviewed human UniProt protein sequences. Supplemental Table 1 and main Figure 1 have been updated after this correction. This error does not change our interpretation of the results.

3. Tables S1: The "#peptides" column. This column does not contain the number of unique peptides identified per protein: It is not possible to identify 889 unique tryptic peptides for myosin 9 (P35579). The

numbers shown might be spectral counts instead. However, if these are spectral counts, it is not clear what the quality filter criterion “a minimum of two matching peptides per protein” means.

We use Integrated Proteomics Pipeline (IP2), developed by the Yates laboratory, for MS/MS analysis. Our IP2 workflow defines each unique chromatogram as a “sequence count”. Different charge states of the same peptide are also counted individually as “sequence counts” by IP2. In the example given – myosin 9 – IP2 scores 889 “sequence counts” and several thousand spectral counts. Because each MS1 chromatograph, or “sequence count” represents an independent data point for SILAC ratio analysis, reporting the total sequence counts is a more informative measure of how many data points were used for SILAC ratio analysis than the number of unique tryptic peptides.

We apologize for this confusion and have modified the “#peptides” column in all Supplemental Tables to read “sequence counts”.

4. Table S1: It is also strange that some proteins were identified with many peptides in wild-type cells and a much smaller number in HGPS cells (for example, P35579 has 889 peptides at 0D in wt but only 90 in HGPDS. In fact, in the latter dataset the coverage was apparently much lower, since only a single protein was identified with more than 100 peptides.

Yes, coverage was especially low in the HGPS 0D dataset.

5. Most of the points above also apply to Table S2

6. There is very little information on how exactly the data was acquired and analyzed. Which resolution, AGS target values, dynamic exclusion settings were used on the Tribrid and the Q-Exactive instrument? How was the HPLC performed (which instrument, gradient, how long)? How was the data calibrated? Is it true that semitryptic peptides were allowed “one tryptic cleavage end per peptide” (which would be problematic)? Also, how exactly were protein computed from peptide ratios?

We have now expanded this section of our Methods section.

7. The authors should upload their raw data to a public resource and make it available for reviewers. Without this it is impossible to assess data quality.

We have uploaded our datasets to the PRIDE repository (www.ebi.ac.uk/pride). To access them, please use the following information and login credentials:

Project Name: SILAC pulse labeling in HGPS fibroblasts
[Please note that this is an incomplete submission; this project will be updated to a complete submission (with supporting mzXML files) shortly.]

Project accession: PXD006015
Project DOI: Not applicable

Username: reviewer45927@ebi.ac.uk
Password: F0jyusaW

Project accession: PXD006016
Project DOI: Not applicable

Username: reviewer01836@ebi.ac.uk
Password: mLbiQIma

Project Name: SILAC analysis of relative protein abundance in HGPS fibroblasts

Project accession: PXD006012
Project DOI: 10.6019/PXD006012

Username: reviewer33123@ebi.ac.uk
Password: 1GADypU7

Project accession: PXD006013
Project DOI: 10.6019/PXD006013

Username: reviewer90113@ebi.ac.uk
Password: sDLANivi

Project accession: PXD006014
Project DOI: 10.6019/PXD006014

Username: reviewer89108@ebi.ac.uk
Password: OfSl8Uj2

8. Fig. 3A: This figure only shows wild-type cell lines. Therefore, it cannot be used to compare rRNA synthesis between HGPS cells and wild-type cells. It is not clear where the data in Fig. 3 B comes from.

This figure was mislabeled and we have corrected the error.

9. Line 63 and legend to Fig 1, "pSILAC": The authors did not use "pSILAC" (as in Schwanhausser et al., Proteomics, 2009 and Selbach et al., Nature, 2008) but dynamic SILAC (as in Doherty et al., JPR, 2009 and Schwanhausser et al., Nature, 2011).

Thank you for pointing out this problem in terminology. We will revert to "SILAC" for clarity and simplicity's sake.

10. The authors used quiescent cells. A potential problem is that in this system, a considerable fraction of amino acids that are incorporated into newly synthesized proteins might be recycled. Importantly, in case aa recycling rates differ between wt and HGPS cells, this would look like differences in protein turnover. Therefore, the authors need to carefully check the relative isotope abundance (RIA) in the aminoacyl tRNA precursor pool for both Lys and Arg. The simplest way of doing this is to look at peptides with missed cleavages.

If recycled amino acids were a major contribution to newly synthesized proteins, we would expect to find peptides with missed cleavages containing various permutations of light/heavy Lys and light/heavy Arg. To check this, we followed your suggestion and compared MS1 spectra for a peptide with a missed cleavage (TKGVDEVTIVNILTNR) from Annexin A2 found in both our WT and HGPS datasets in samples subjected to 6 days of labeling with heavy Lys and Arg (please see Reviewer Figure 2). In the WT sample, a major peak corresponding to the light (old) peptide and a slightly more abundant peak corresponding to the heavy (new) peptide were found. Intermediate peaks containing heavy Lys / light Arg and heavy Arg / light Lys were detectable, but were much less abundant. When we analyzed the same peptide in the HGPS sample, the most abundant peak was the heavy (new) peptide, and the light (old) peptide was much less abundant, as one would expect if the protein were turning over more rapidly in HGPS cells. Intermediate mixed peaks were again detectable but minor, and importantly, were not any more abundant than in the corresponding WT sample. From this we conclude that amino acid recycling in quiescent HGPS cells is unlikely to be a confounding factor in interpreting our SILAC time course data.

11. Line 74 "Importantly, Histone H3.1, which is not synthesized in quiescent cells25 incorporated little label in normal and HGPS samples (Fig. 1h), indicating that both cell populations maintained quiescence effectively." I agree that this result is consistent with the idea that cells are largely quiescent. However, there is some label incorporation, indicating that the cells are not fully quiescent. Moreover, since even a small increase in the fraction of dividing HGPS cells would lead to an increase in the apparent protein "turnover", this aspect must be checked more thoroughly. For example, the authors could use BrdU labeling to carefully check if there is really no cell division (and, most importantly, no difference).

We have performed this experiment, and it indicates that WT and HGPS cells maintain quiescence to an equivalent extent. These data are now included in Supplementary Figure 1.

12. Fig. 4 A is not a volcano plot. A volcano plot displays the fold change (on the x axis) against the significance of a change (on the y axis, typically as negative log p-values). Fig. 4 A shows the number of peptides on the y axis, which has nothing to do with the significance of observed changes.
We have removed this erroneous description.

13. Legend to Fig. 4: “Deviation from a normal distribution confirmed by D’Agostino and Pearson normality test” (also main text, line 135): SILAC-based log2-fold changes in protein abundance are usually not normally distributed. This is even the case when two untreated samples are compared with each other. Therefore, the statistical test is meaningless.
We have removed this from our analysis.

Reviewers' Comments:

Reviewer #1:
Remarks to the Author:
The new version of the manuscript by Buchwalter and Hetzer has not addressed convincingly any of my main criticisms to the original manuscript.
1. The work is based exclusively on the use of cultured cells, without any data supporting the relevance of their findings in patients or in animal models.
2. The authors do not provide any clue about the mechanisms by which progerin or lamin A deficiency induce the alleged nucleolar expansion and ribosome biogenesis.
3. The manuscript does not explain how the increased protein biosynthesis is balanced to lead to increased protein turnover in the absence of protein degradation. In the rebuttal letter, the authors suggest that “HGPS cells cope with increased protein output by dividing more rapidly”. However, in the main text they indicate that they have analyzed non-dividing quiescent cells (lines 67-68), which makes that explanation senseless. On the other hand, if an increased proliferation rate were a valid explanation for of the increased protein turnover reported the novelty of their findings would be questionable: shouldn’t increased cell proliferation necessarily involve increased protein synthesis?
4. I disagree with the authors regarding the need of discussing the results derived from other experimental systems, as discussed in points 4 and 5 of the original review, even when those results do not necessarily fit in their model.
5. In their response to point 7, the authors state that analyzing whether age-associated nucleolar changes are accompanied of more active protein synthesis “exceeds the scope of the current study”. In my opinion, this rather simple study would be essential to support the relevance of the part of the manuscript related with normal aging.

Reviewer #2:
Remarks to the Author:
The comments to the authors from the four reviewers are wide-ranging and comments have been restricted to the authors’ attempts to address the concerns from this reviewer. In general, they have been adequately met.

There is some disappointment that no attempt was made to assess the number of rDNA repeats and while normal variation might make these measures hard to assess significantly in the number of cell lines tested, the attempt to measure this should be made since it seems directly germane to the relevant conclusions of the manuscript.

One comment made by another reviewer expressed surprise that the mTOR pathway was not upregulated based on findings in Lmna-/- mice. The finding in this paper and is not contradictory since the Lmna-/- model is not directly comparable to progerin mice and it is not clear that the same result should be expected. If anything, one would not expect elevated mTOR signaling based on published studies in Zmpste24-/-.

Reviewer #3:
Remarks to the Author:
The authors have only addressed my comments partially (pre-rRNA processing data).

-I still believe that 'expansion' is a bad choice of term.

In the field, we regularly use the term 'extension' (for ribosomal proteins) and 'expansion' (for ribosomal RNAs). 'Enlargement' would be better here but, of course, this is at the authors'

discretion.

-When the authors refer to Badertscher et al Cell Rep (2015) to discuss the assembly factors; they should also refer to Tafforeau et al Molecular Cell (2013) who provided the first comprehensive list of processing factors in human cells.

Reviewer #4:
Remarks to the Author:
First, I would like to apologize to the authors for taking so long to review their revised manuscript -- I simply couldn't manage to get this done sooner.

The authors have addressed most of the points I raised. However, there are still three points that remain unclear (see below). The first point is particularly critical: The impact of the mutation on protein synthesis, degradation, secretion and cell division are still not clear to me. I find the new EdU experiment and its interpretation confusing.

1. The authors conclude that HGPS cells show increased translation without notable changes in protein degradation or cell division. (line 89, “we conclude that increased turnover of the nuclear proteome in HGPS (Fig. 1) is due to elevated synthesis of new protein, and not to increased degradation of old protein”). The problem with this statement is that enhanced protein synthesis without increasing protein degradation or cell division would lead to a continuously increasing protein content per cell. Thus, HGPS cells would never reach a steady state and continuously produce more protein. I cannot imagine how this is possible: Either the cell division rate or protein degradation (or both) must also increase. The only other possibility that comes to mind is that HGPS cells might secrete more proteins than wt cells.

Our data suggest that total protein content (including secreted protein output) is elevated in HGPS. Based on a study by Bridger and Kill (Experimental Gerontology, 2004) describing an early “hyperproliferative” phase in HGPS fibroblasts followed by premature senescence, and our own observations, we expect that HGPS cells cope with increased protein output by dividing more rapidly, at least at earlier passages (which is when all our analyses were performed). We have performed an EdU incorporation experiment that confirms this. This data is now included in Supplementary Figure 1.Our data suggest that total protein content (including secreted protein output) is elevated in HGPS.

I don’t understand this. On the one hand, the authors argue that cells are not dividing (“Importantly, Histone H3.1, which is not synthesized in quiescent cells incorporated little label in normal and HGPS samples (Fig. 1h), and quiescent cells do not incorporate EdU (Supplementary Fig. 1b) indicating that both cell populations maintain quiescence effectively.”) On the other hand, they now state that HGPS cells cope with increased protein output by dividing more rapidly. However, the experiment in Fig. S1B shows no significant increase in EdU incorporation in quiescent HG-1 cells. I do not understand how the EdU experiment confirms the hypothesis that HG-1 cells divide more rapidly.

4. Table S1: It is also strange that some proteins were identified with many peptides in wild-type cells and a much smaller number in HGPS cells (for example, P35579 has 889 peptides at 0D in wt but only 90 in HGPDS. In fact, in the latter dataset the coverage was apparently much lower, since only a single protein was identified with more than 100 peptides.

Yes, coverage was especially low in the HGPS 0D dataset.

The vastly different coverage is quite worrying. How can the authors rule out that this impacts their quantification?

6. There is very little information on how exactly the data was acquired and analyzed. Which resolution, AGS target values, dynamic exclusion settings were used on the Tribrid and the Q-Exactive instrument? How was the HPLC performed (which instrument, gradient, how long)? How was the data calibrated? Is it true that semitryptic peptides were allowed “one tryptic cleavage end per peptide” (which would be problematic)? Also, how exactly were protein computed from peptide ratios?

We have now expanded this section of our Methods section.

The new information provided indicates that the authors used suboptimal settings for their mass spectrometric analysis. Most importantly, the MS2 AGS target value was 5E6 (which is very high) with a maximum fill time of 500 ms (which is very long). With the top 10 method used this will result in very long cycle times and therefore limit accuracy for quantification (few MS1 peaks / time). Moreover, these settings will also result in under sampling. I don’t understand why these awkward settings were used.

From the editor
As you can see from the comments below, reviewers #1 and #4 continue to find the main hypothesis problematic and insufficiently supported by the data; for example, they point out that cells appear to be quiescent in some experiments, which is difficult to reconcile with the idea that hyperproliferation is the mechanism to cope with the increased protein production in the absence of changes to protein degradation in these cells. It will be essential for publication that you find a way to reconcile your data with the central hypothesis, providing new experimental data if needed to support it, and present a hypothesis that is conceptually acceptable to our reviewers.

The central hypothesis of our paper is this: that mutation to lamin A leads to de-repression of ribosomal DNA, which allows more production of ribosomes, more translation of protein, and contributes to the premature aging phenotype. This is supported by our findings that (1) HGPS cells have less rDNA methylation (Figure 3D,E; Table 1); (2) produce more rRNA (Figure 3A-C); (3) make more ribosomal proteins (Figure 4, Supplemental Figure 7); and (4) have increased protein synthesis output (Figure 2A). We demonstrate that ectopic expression of progerin (Figure 5) or depletion of normal lamin A (Figure 6) causes expansion of nucleoli, which suggests a direct relationship between lamin A and regulation of nucleolar activity.
Excitingly, we find evidence for a general relationship between nucleolar activity and aging by showing that (i) nucleolar size and (ii) rRNA abundance increase with age. This suggests that nucleolar size is a novel biomarker of aging, and this finding is supported and complemented by parallel work from the Antebi group that demonstrates that nucleolar size is predictive of lifespan in C. elegans.

Experiments where cells are quiescent versus cycling. We apologize for not defining these conditions more clearly in the original manuscript, which caused confusion among reviewers about our conclusions. The sole experiments performed in quiescent cells were the SILAC labeling experiments.
Importantly, all non-proteomic experiments that link progerin to nucleolar dysfunction were performed in cycling cells. To test our initial hypothesis that progerin accumulates in HGPS cells, we wanted to track protein turnover by SILAC labeling over time without the confounding factor of cellular turnover. To achieve this, sub-confluent cultures were grown in low serum conditions for several days before analysis. We verified cell cycle exit by performing an EdU labeling time course, shown in Supplemental Figure 1B. This was important to demonstrate that changes in proteome turnover observed in HGPS were not due to changes to the cell cycle. We are confident in the finding that ribosomal proteins are more highly expressed in HGPS (Figure 4), since we also see elevated levels of ribosomal protein S6 in cycling HGPS cells (Supplementary Figure 4), increased translating polysomes in cycling HGPS cells (Supplementary Figure 3), and increased global protein synthesis rates in cycling HGPS cells (Figure 2). An EdU labeling time course in these cycling cell populations indicated that HGPS cells proliferate more rapidly than normal wild type cells. Based on this finding, we suggest that cycling HGPS cells cope with increased protein production by dividing more rapidly. This is consistent with a previous report (Bridger and Kill, Exp Gerontol 2004), which we reference in the text.
For clarity, we have split the quiescent EdU labeling experiment and the cycling EdU labeling experiment into separate figure panels in Supplementary Figure 1. We also emphasize the transition from quiescent to cycling cell cultures more clearly in the text.

We have no problem with the use of cultured cells or absence of mechanism (points 1+2 from reviewer #1), but ask that points 4+5 of reviewer #1 are addressed textually with additional discussion in the manuscript (as you did in the point by point response). We also expect you to explicitly state the limitations of your study, ie that the absence of the experiments suggested by reviewers #2 and #3 is acknowledged. Finally, we strongly recommend that you address the concerns about the mass spec data raised by #4 experimentally, if at all possible; at the very least, these shortcomings should also be discussed.

We have made the requested changes in the manuscript text; please also see responses to reviewers below.

We would therefore ike to give you one more opportunity to revise and resubmit your manuscript, taking into account the points raised. Please highlight all changes in the manuscript text file.

Reviewer #1 (Remarks to the Author):

The new version of the manuscript by Buchwalter and Hetzer has not addressed convincingly any of my main criticisms to the original manuscript.

1. The work is based exclusively on the use of cultured cells, without any data supporting the relevance of their findings in patients or in animal models.
We have used primary human fibroblasts from progeria patients at early passage. While cell culture has its limitations, it has been a good model system for defining the cell biological effects of lamin A mutation on nucleolar activity. Note that complementary work from the Antebi group in C elegans and mammals supports our finding that there is a direct relationship between nucleolar size/activity and age.

2. The authors do not provide any clue about the mechanisms by which progerin or lamin A deficiency induce the alleged nucleolar expansion and ribosome biogenesis.
Our model (Figure 7c) summarizes our current thinking on this: that mutation to lamin A leads to de-repression of ribosomal DNA, which allows more production of ribosomes, more translation of protein, and contributes to the premature aging phenotype. This is supported by our findings that (1) HGPS cells have less rDNA methylation (Figure 3D,E; Table 1); (2) produce more rRNA (Figure 3A-C); (3) make more ribosomal proteins (Figure 4, Supplemental Figure 7); and (4) increased protein synthesis output (Figure 2A). We demonstrate that ectopic expression of progerin (Figure 5) or depletion of normal lamin A (Figure 6) causes expansion of nucleoli, which suggests a direct relationship between lamin A and regulation of nucleolar activity. Future work will define the specific links between lamin A and chromatin state of rDNA.

3. The manuscript does not explain how the increased protein biosynthesis is balanced to lead to increased protein turnover in the absence of protein degradation. In the rebuttal letter, the authors suggest that "HGPS cells cope with increased protein output by dividing more rapidly". However, in the main text they indicate that they have analyzed non-dividing quiescent cells (lines 67-68), which makes that explanation senseless. On the other hand, if an increased proliferation rate were a valid explanation for of the increased protein turnover reported the novelty of their findings would be questionable: shouldn't increased cell proliferation necessarily involve increased protein synthesis?
Please see our explanation of this above under our comments to the editor.
In regard to the relationship between protein synthesis and cell proliferation: the fact that progerin expression or lamin A depletion rapidly and potently increase nucleolar size indicates a novel relationship between lamin A and nucleolar regulation that in turn affects protein synthesis rates. This is one of the core novel findings of our paper.

4. I disagree with the authors regarding the need of discussing the results derived from other experimental systems, as discussed in points 4 and 5 of the original review, even when those results do not necessarily fit in their model.
We have added discussion of mTOR signaling in laminopathy models and rDNA methylation analysis in the Zmpste24 -/- mouse as requested. As reviewer #1 pointed out, Lmna -/- mice exhibit elevated mTOR signaling in cardiac tissue (Ramos et al., Sci Translational Med 2012) and adipose tissue (Liao et al., Cell Reports 2016). As mTOR promotes protein synthesis, one might expect that elevated mTOR signaling could drive the increase in protein synthesis that we observed in HGPS. We did not find this to be the case (Supplementary Figure 4). Rather, our findings are consistent with a previous analysis of mTOR signaling in patient-derived HGPS cultures (Cao et al., Sci Transl Med 2011). We now discuss this more extensively in the text as we describe our analysis of mTOR signaling.
As reviewer #1 brought to our attention, there is one previous study addressing rDNA methylation status in the Zmpste24 -/- mouse, a premature aging model (Osorio et al Aging Cell 2010). rDNA methylation has not been analyzed in other models of HGPS to our knowledge. As alluded to by reviewer #2, extrapolating results from the Lmna -/- and Zmpste24 -/- mouse models to human HGPS is difficult, as there are several instances of contradictory results between these two models. For instance, mTOR signaling is elevated in the Lmna -/- mouse (citations above), but decreased in the Zmpste24 -/- mouse (Marino et al., Hum Mol Genet 2008).

Importantly, these knockout mice are genetically dissimilar to human HGPS, which is caused by a heterozygous point mutation in Lmna. The recent development of knock-in mice expressing the human HGPS allele (Osorio et al Sci Transl Med 2011) is an improvement in this regard, but phenotypes relevant to our study have not been reported in this model.
With that caveat noted, Osorio et al. (Aging Cell, 2010) reported that regions of rDNA loci are more heavily methylated in Zmpste24 -/- mice. An important distinction between that study and ours is that we specifically analyzed the methylation status of the rDNA promoter region, where de-methylation has been shown to directly correlate with Pol I transcription in human cells (Brown and Szyf, Mol Cell Biol, 2007). In the Osorio et al., study, rDNA methylation status changed little within the promoter region, but there were much more pronounced differences internally within the rDNA gene. It is unclear how internal methylation correlates with the transcriptional activity of rDNA loci.
We now include discussion of these results and the relevant citations in the manuscript (Discussion section) as requested.

5. In their response to point 7, the authors state that analyzing whether age-associated nucleolar changes are accompanied of more active protein synthesis “exceeds the scope of the current study”. In my opinion, this rather simple study would be essential to support the relevance of the part of the manuscript related with normal aging.
Work from C elegans indicates that there is a trend towards more protein synthesis with age (Vukoti et al., J Proteome Res 2015), and we intend to analyze this in human cells in future work. Both our manuscript and Tiku et al. demonstrate a relationship between nucleolar activity and age; Tiku et al. also did not analyze changes to protein synthesis with age. Importantly, elevated ribosome biogenesis itself could have profound effects on cellular energy stores and thus could contribute to aging phenotypes. Incomplete or imbalanced induction of ribosome biogenesis can lead to ribosome quality control-related stress. These are possible outcomes beyond protein synthesis being elevated in the course of the normal aging process. We have extended our discussion of this in the text.

Reviewer #2 (Remarks to the Author):

The comments to the authors from the four reviewers are wide-ranging and comments have been restricted to the authors’ attempts to address the concerns from this reviewer. In general, they have been adequately met.

There is some disappointment that no attempt was made to assess the number of rDNA repeats and while normal variation might make these measures hard to assess significantly in the number of cell lines tested, the attempt to measure this should be made since it seems directly germane to the relevant conclusions of the manuscript.
We intend to analyze this in the future, however it may be necessary to generate a much larger number of human samples than we currently have access to in order to make accurate assessments of rDNA copy number changes, based on extensive variation across the population (Gibbons et al, Nature Communications 2016). We have added discussion of this in the text.

One comment made by another reviewer expressed surprise that the mTOR pathway was not upregulated based on findings in Lmna-/- mice. The finding in this paper and is not contradictory since the Lmna-/- model is not directly comparable to progerin mice and it is not clear that the same result should be expected. If anything, one would not expect elevated mTOR signaling based on published studies in Zmpste24-/-.
Thank you for pointing this out. We have added discussion of previous work on mTOR signaling in laminopathy models in the text.

Reviewer #3 (Remarks to the Author):

The authors have only addressed my comments partially (pre-rRNA processing data).

-I still believe that 'expansion' is a bad choice of term.

In the field, we regularly use the term 'extension' (for ribosomal proteins) and 'expansion' (for ribosomal RNAs). 'Enlargement' would be better here but, of course, this is at the authors' discretion.
We will proceed with ‘expansion’.

-When the authors refer to Badertscher et al Cell Rep (2015) to discuss the assembly factors; they should also refer to Tafforeau et al Molecular Cell (2013) who provided the first comprehensive list of processing factors in human cells.
Thank you for pointing this out; we have added a reference to this earlier paper. We did not find any factors identified only by Tafforeau et al. in our datasets.

Reviewer #4 (Remarks to the Author):

First, I would like to apologize to the authors for taking so long to review their revised manuscript -- I simply couldn't manage to get this done sooner.

The authors have addressed most of the points I raised. However, there are still three points that remain unclear (see below). The first point is particularly critical: The impact of the mutation on protein synthesis, degradation, secretion and cell division are still not clear to me. I find the new EdU experiment and its interpretation confusing.

1. The authors conclude that HGPS cells show increased translation without notable changes in protein degradation or cell division. (line 89, “we conclude that increased turnover of the nuclear proteome in HGPS (Fig. 1) is due to elevated synthesis of new protein, and not to increased degradation of old protein”). The problem with this statement is that enhanced protein synthesis without increasing protein degradation or cell division would lead to a continuously increasing protein content per cell. Thus, HGPS cells would never reach a steady state and continuously produce more protein. I cannot imagine how this is possible: Either the cell division rate or protein degradation (or both) must also increase. The only other possibility that comes to mind is that HGPS cells might secrete more proteins than wt cells.

Our data suggest that total protein content (including secreted protein output) is elevated in HGPS. Based on a study by Bridger and Kill (Experimental Gerontology, 2004) describing an early “hyperproliferative” phase in HGPS fibroblasts followed by premature senescence, and our own observations, we expect that HGPS cells cope with increased protein output by dividing more rapidly, at least at earlier passages (which is when all our analyses were performed). We have performed an EdU incorporation experiment that confirms this. This data is now included in Supplementary Figure 1.Our data suggest that total protein content (including secreted protein output) is elevated in HGPS.

I don’t understand this. On the one hand, the authors argue that cells are not dividing (“Importantly, Histone H3.1, which is not synthesized in quiescent cells incorporated little label in normal and HGPS samples (Fig. 1h), and quiescent cells do not incorporate EdU (Supplementary Fig. 1b) indicating that both cell populations maintain quiescence effectively.”) On the other hand, they now state that HGPS cells cope with increased protein output by dividing more rapidly. However, the experiment in Fig. S1B shows no significant increase in EdU incorporation in quiescent HG-1 cells. I do not understand how the EdU experiment confirms the hypothesis that HG-1 cells divide more rapidly.
We apologize for not defining our experimental conditions more clearly. Importantly, SILAC experiments were performed in non-dividing cells, and all non-proteomic experiments that link progerin to nucleolar dysfunction were performed in cycling cells. Please see our response to the editor above for further clarification.

4. Table S1: It is also strange that some proteins were identified with many peptides in wild-type cells and a much smaller number in HGPS cells (for example, P35579 has 889 peptides at 0D in wt but only 90 in HGPDS. In fact, in the latter dataset the coverage was apparently much lower, since only a single protein was identified with more than 100 peptides.

Yes, coverage was especially low in the HGPS 0D dataset.

The vastly different coverage is quite worrying. How can the authors rule out that this impacts their quantification?
The day 0 sample with the lowest coverage is only a control for the starting point of the experiment (when no label is present). Data from the subsequent time points are not normalized or referenced to the values from day 0, since SILAC is an internally normalized, ratiometric method. For all data analysis, we rigorously filtered and selected only spectra of high quality (with a profile score above a cutoff value of 0.8, see Methods) for quantitative analysis of SILAC label incorporation. Only proteins identified with at least 2 peptides were included in the dataset shown in Figure 1 and Table S1. In SILAC experiments, each peptide contains quantitative ratiometric information about label incorporation, and each peptide is thus an independent data point. By selecting only spectra that pass rigorous quality control filters, then pooling multiple peptide spectra per protein, we can be confident in the label incorporation data shown. (see Reviewer Figure for an example.) For this analysis, we followed published protocols from experts in SILAC-MS (see Ong & Mann, Nat Protocols 2006) and expertise within our own group (see Toyama et al., Cell 2013). We have cited these papers in our Methods section.

6. There is very little information on how exactly the data was acquired and analyzed. Which resolution, AGS target values, dynamic exclusion settings were used on the Tribrid and the Q-Exactive instrument? How was the HPLC performed (which instrument, gradient, how long)? How was the data calibrated? Is it true that semitryptic peptides were allowed “one tryptic cleavage end per peptide” (which would be problematic)? Also, how exactly were protein computed from peptide ratios?

We have now expanded this section of our Methods section.

The new information provided indicates that the authors used suboptimal settings for their mass spectrometric analysis. Most importantly, the MS2 AGS target value was 5E6 (which is very high) with a maximum fill time of 500 ms (which is very long). With the top 10 method used this will result in very long cycle times and therefore limit accuracy for quantification (few MS1 peaks / time). Moreover, these settings will also result in under sampling. I don’t understand why these awkward settings were used.
These instrument settings were previously optimized for obtaining high quality MS2 spectra of low-abundance proteins, and our colleague Alan Saghatelian recently published a paper using these settings (Ma et al., Anal Chem 2016). To demonstrate that we achieved sufficient coverage of proteins of interest to draw conclusions from our data, we are sharing MS data from two proteins relevant to our conclusions: 60S ribosomal protein L5 (UniProt ID P46777), and 40S ribosomal protein S8 (UniProt ID P62241). We detected RPL5 with 23 unique peptides, and RPS8 with 16 unique peptides. These peptides were detected ranging from 6 to 18 times in MS1 scans (please see Reviewer Table).
Further, the equipment settings critiqued were only used on a Q Exactive MS instrument. Data from this machine appears only in Figure 4b-c; all other MS experiments were performed on a Fusion TriBrid MS instrument, using settings that were not critiqued by the reviewer. We performed a very similar experiment to the data that appears in Figure 4b-c on an additional pair of WT and HGPS cell lines that appears in Supplemental Figure 7. These samples were run on the Fusion MS instrument. There is a minor increase in proteome coverage; ~900 proteins were identified with at least 3 peptides in Figure 4b-c, and ~1000 proteins were identified with at least 3 peptides in Supplementary Figure 7. Importantly, the experimental outcomes in Supplemental Figure 7 are consistent with those reported in Figure 4: that ribosomal proteins are more abundant in HGPS cells.
We have added a reference to Dr. Saghatelian’s recent paper to our Methods section, and specified more clearly which MS machines were used to generate data in each figure panel.

Reviewer Figure

Example:
isocitrate dehydrogenase

Example chromatograms for the indicated tryptic peptide from 1, 2, and 3 days of a SILAC labeling time course. The heavy peptide labeling new proteins is shown in red; the light peptide labeling old proteins is shown in blue.

To quantify peptide turnover at each time point, the area under each curve is integrated. These areas are then ratioed to each other. This ratio directly reports the relative abundance of the "old" (blue) and "new" (red) protein at each time point. These values were computed for all peptides detected for a protein of interest, then averaged to obtain protein turnover rates.

R.FKDIFQEIFDK

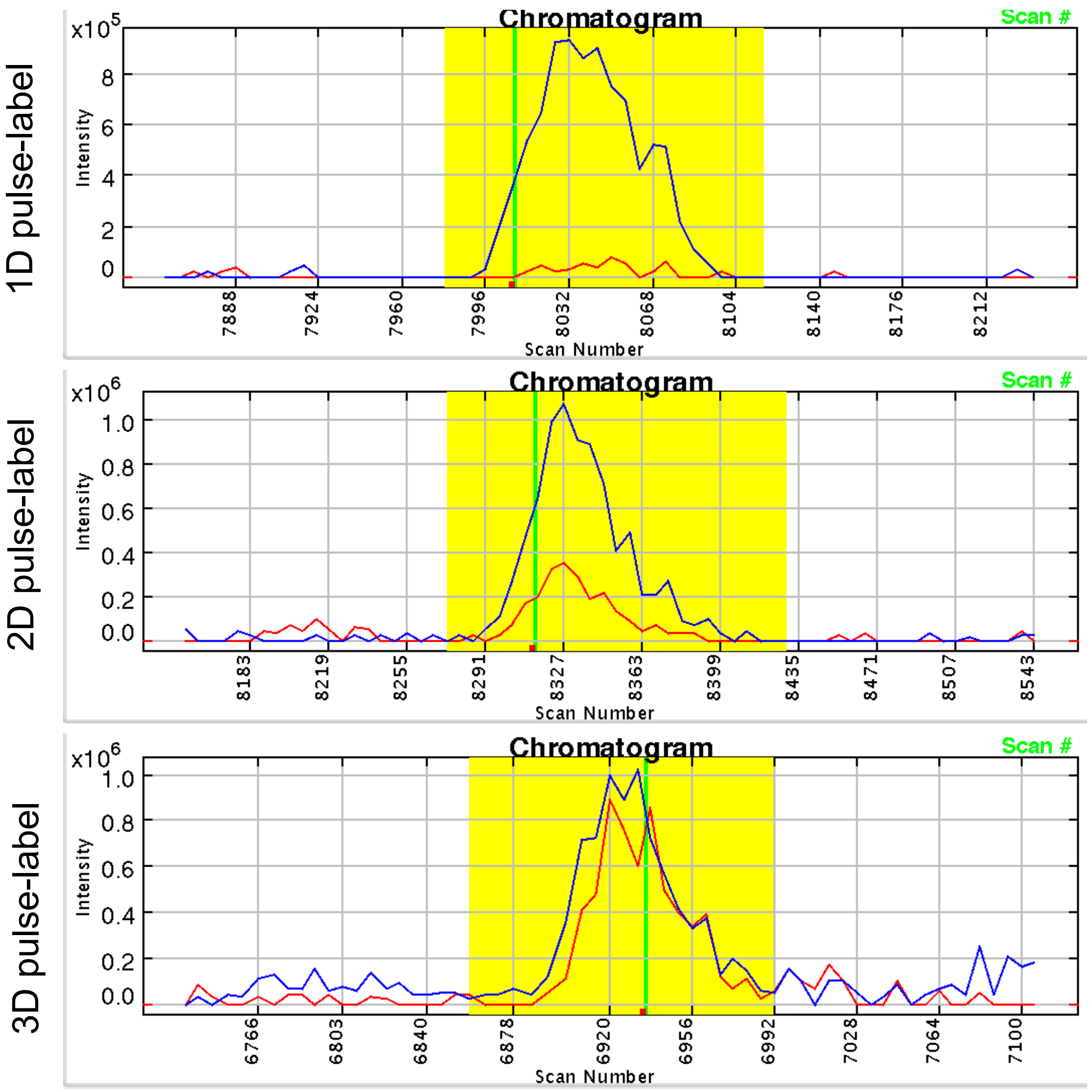

Unique	Sequence	UniProt ID	Scan	charge state	Protein Description	# MS1 measurements
TRUE	R.VTNRDIICQIAYAR.I	[P46777]	18241	3	[P46777 60S ribosomal protein L5	6
TRUE	R.DIICQIAYAR.I	[P46777]	19398	2	[P46777 60S ribosomal protein L5	9
TRUE	R.DIICQIAYAR.I	[P46777]	19371	2	[P46777 60S ribosomal protein L5	9
TRUE	R.IEGDMIVCAAYAHELI	[P46777]	20737	3	[P46777 60S ribosomal protein L5	12
TRUE	R.IEGDMIVCAAYAHELI	[P46777]	20755	3	[P46777 60S ribosomal protein L5	12
TRUE	K.VGLTNYAAAYCTGLLI	[P46777]	26207	2	[P46777 60S ribosomal protein L5	12
TRUE	K.VGLTNYAAAYCTGLLI	[P46777]	26172	3	[P46777 60S ribosomal protein L5	12
TRUE	K.GAVDGGLSIPHSTK.R	[P46777]	13269	2	[P46777 60S ribosomal protein L5	14
TRUE	K.GAVDGGLSIPHSTK.R	[P46777]	13262	3	[P46777 60S ribosomal protein L5	14
TRUE	K.GAVDGGLSIPHSTK.R	[P46777]	13245	2	[P46777 60S ribosomal protein L5	14
TRUE	K.GAVDGGLSIPHSTK.R	[P46777]	13263	3	[P46777 60S ribosomal protein L5	14
TRUE	K.RFPGYDSESK.E	[P46777]	7994	2	[P46777 60S ribosomal protein L5	18
TRUE	K.RFPGYDSESK.E	[P46777]	7965	2	[P46777 60S ribosomal protein L5	18
TRUE	K.EFNAEVHR.K	[P46777]	5879	2	[P46777 60S ribosomal protein L5	18
TRUE	K.HIMGQNVADYMR.Y	[P46777]	15454	3	[P46777 60S ribosomal protein L5	12
TRUE	K.HIMGQNVADYMR.Y	[P46777]	15456	3	[P46777 60S ribosomal protein L5	12
TRUE	R.YLMEEDEDAYKK.Q	[P46777]	12025	2	[P46777 60S ribosomal protein L5	12
TRUE	R.YLMEEDEDAYKK.Q	[P46777]	12020	3	[P46777 60S ribosomal protein L5	12
TRUE	R.YLMEEDEDAYKK.Q	[P46777]	12044	2	[P46777 60S ribosomal protein L5	12
TRUE	R.YLMEEDEDAYKK.Q	[P46777]	12013	3	[P46777 60S ribosomal protein L5	12
TRUE	K.QFSQYIK.N	[P46777]	12598	2	[P46777 60S ribosomal protein L5	9
TRUE	K.NSVTPDMMEEMYK.	[P46777]	19275	2	[P46777 60S ribosomal protein L5	12
TRUE	K.NSVTPDMMEEMYK.	[P46777]	19282	2	[P46777 60S ribosomal protein L5	12

Unique	Sequence	Proteins	Scan	charge state	Protein Description	# MS1 measurements
TRUE	K.YELGRPAANTK.I	[P62241]	7725	2	[P62241 40S ribosomal protein S8	11
TRUE	R.LDVGNFSWGSECCTF	[P62241]	20962	2	[P62241 40S ribosomal protein S8	11
TRUE	R.LDVGNFSWGSECCTF	[P62241]	20967	2	[P62241 40S ribosomal protein S8	11
TRUE	R.IIDVVYNASNNELVR.	[P62241]	20457	2	[P62241 40S ribosomal protein S8	10
TRUE	R.IIDVVYNASNNELVR.	[P62241]	20469	2	[P62241 40S ribosomal protein S8	10
TRUE	K.NCIVLIDSTPYR.Q	[P62241]	19894	2	[P62241 40S ribosomal protein S8	11
TRUE	R.QWYESHYALPLGR.K	[P62241]	19920	3	[P62241 40S ribosomal protein S8	14
TRUE	K.LTPEEEEILNK.K	[P62241]	15529	2	[P62241 40S ribosomal protein S8	12
TRUE	K.LTPEEEEILNK.K	[P62241]	15539	2	[P62241 40S ribosomal protein S8	12
TRUE	K.ISSLLEEQFQQGK.L	[P62241]	18570	2	[P62241 40S ribosomal protein S8	11
TRUE	K.ISSLLEEQFQQGK.L	[P62241]	18582	2	[P62241 40S ribosomal protein S8	11
TRUE	K.LLACIASRPGQCGR.A	[P62241]	12876	3	[P62241 40S ribosomal protein S8	12
TRUE	R.ADGYVLEGK.E	[P62241]	10112	2	[P62241 40S ribosomal protein S8	12
TRUE	R.ADGYVLEGK.E	[P62241]	10122	2	[P62241 40S ribosomal protein S8	12
TRUE	K.ELEFYLR.K	[P62241]	20073	2	[P62241 40S ribosomal protein S8	9
TRUE	K.ELEFYLR.K	[P62241]	20091	2	[P62241 40S ribosomal protein S8	9

Reviewers' Comments:

Reviewer #4:
Remarks to the Author:
The authors now explain that they only did the first experiment in quiescent cells and all others in cycling cells.

I can understand now that in cycling cells the increased protein synthesis can be compensated by increased proliferation. However, it is not clear to me what happens in quiescent cells: As explained before, there is no way how cells can increase protein synthesis (over extended periods of time) without also increasing degradation/secretion or proliferation. Since most cells in an organism are quiescent, this is probably the most relevant state of cells for aging. So what happens in quiescent cells?

REVIEWERS' COMMENTS:

Reviewer #4 (Remarks to the Author):

The authors now explain that they only did the first experiment in quiescent cells and all others in cycling cells.

I can understand now that in cycling cells the increased protein synthesis can be compensated by increased proliferation. However, it is not clear to me what happens in quiescent cells: As explained before, there is no way how cells can increase protein synthesis (over extended periods of time) without also increasing degradation/secretion or proliferation. Since most cells in an organism are quiescent, this is probably the most relevant state of cells for aging. So what happens in quiescent cells?

The reviewer states that "there is no way how cells can increase protein synthesis (over extended periods of time) without also increasing degradation/secretion or proliferation". We did not analyze cells maintained in quiescence for long periods of time; we used cells maintained in quiescence for up to 6 days in proteomic experiments strictly for technical reasons, as we attempted to clarify in our previous response. This means that we observed elevated proteome turnover (Figure 1) as well as increased abundance of ribosomal proteins (Figure 4) in quiescent cells. We also observed increased abundance of ribosomal protein S6 in Western blots in cycling cells (Figure S4) and in quiescent cells (not shown), giving us confidence that increased ribosome biogenesis and protein synthesis are phenotypes occurring in both non-dividing and dividing progeroid cells. We expect that de-regulation of these processes will profoundly affect cellular energy metabolism in both non-dividing and dividing states.

While it is true that a large proportion of cells in an organism are non-dividing, there is a consensus in the aging field that changes to the function of proliferating cell populations are a major contributor to age-associated decline of tissues and organs (see Lopez-Otin et al., Cell, 2013). For example, the ability of stem cell pools to proliferate in order to repair tissues declines with age (see Liu and Rando, J Cell Biol 2011). Replicative senescence of cells in proliferative tissues also contributes to age-associated decline, as senescent cells secrete pro-inflammatory proteins and have paracrine effects on tissue function (see Campisi, Annu Rev Physiology, 2013). Importantly, HGPS exhibits both stem cell exhaustion (see Scaffidi & Misteli, Nat Cell Biol 2008) and accelerated senescence onset (see Kudlow et al., Mol Biol Cell 2008), and we think that the cellular phenotype we have uncovered is likely to be a driving factor in these organismal phenotypes.

Importantly, our study is the first evidence that increased protein synthesis occurs in premature aging, and that nucleolar activity increases as aging progresses. We also demonstrate first evidence for a link between lamin A and regulation of ribosome biogenesis. While the question of how cells cope with increased ribosome biogenesis and protein synthesis is an intriguing one, we assert that ribosome biogenesis is an attractive therapeutic target in progeria and in normal aging regardless of what specifically these secondary "coping mechanisms" are.
